# Redox Homeostasis as a Key Regulator of Intramolecular Cyclization in Fungal Perylenequinones

**DOI:** 10.1021/acschembio.5c00369

**Published:** 2025-08-12

**Authors:** Reema A. Al-Qiam, Firoz S. T. Khan, Huzefa A. Raja, Tyler N. Graf, Cedric J. Pearce, Nicholas H. Oberlies, Shabnam Hematian

**Affiliations:** † Department of Chemistry and Biochemistry, 14616 University of North Carolina at Greensboro, Greensboro, North Carolina 27402, United States; ‡ 331581 Mycosynthetix, Inc., Hillsborough, North Carolina 27278, United States; § Department of Chemistry, Virginia Tech, Blacksburg, Virginia 24061, United States

## Abstract

Perylenequinones (PQs) such as hypocrellins and hypomycins are fungal-derived redox-active metabolites with known roles as photosensitizers in the oxidative stress response and applications in photodynamic therapy (PDT). Here, we report that *Shiraia* sp., a filamentous fungus, can survive and grow under strictly anaerobic (argon) conditions—an unexpected finding for a multicellular eukaryote. Modulating redox homeostasis through chemical reduction and oxygen limitation promotes the intramolecular cyclization of hypocrellins, enhancing hypomycin biosynthesis. Moisture content further influences these transformations, with high water levels favoring keto–enol tautomerization and dry, reducing environments promoting hydride substitution at the peripheral positions. These findings highlight redox modulation as a key driver of perylenequinone metabolism and suggest that PQs may contribute to maintaining redox balance under anaerobic stress, hinting at a broader role in oxygen-independent adaptation in filamentous fungi. This work offers new insights at the interface of redox biology, chemical signaling, and fungal metabolism, with potential implications for the stability and function of PQ-based PDT agents in hypoxic, reducing conditions such as tumor microenvironments.

## Graphical abstract

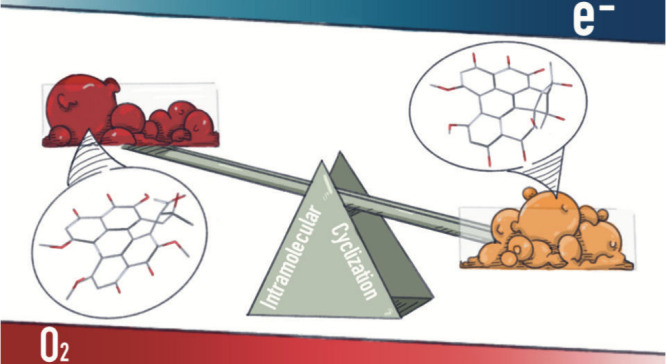

Perylenequinones are naturally occurring organic dyes characterized by their unique chemical structure and diverse biological properties.^1^ They were initially identified for their phototoxic effects on plants.^1,2^ Fungi serve as the primary source of perylenequinones, accounting for ∼ 85% of all reported analogues.^3,4^ Among these, hypocrellins—a notable group of fungal perylenequinones with a pentacyclic, axially chiral core—have garnered attention in photodynamic therapy (PDT) due to their efficacy as photosensitizers.^5–8^ Upon light exposure, hypocrellins generate highly cytotoxic singlet oxygen (^1^O_2_) through energy transfer, leading to the destruction of cancer cells or various pathogens.^2,6^ Compared to conventional treatments, these compounds exhibit strong photodynamic activity with minimal dark toxicity and a lower risk of resistance development.^1,3,6,9^ Despite challenges, such as limited light penetration and oxygen dependence, ongoing PDT research focuses on improving the delivery and stability of hypocrellins, positioning them as promising candidates for cancer and infectious disease treatment.^7,8,10–13^ Given their biological relevance, investigating the metabolic transformations of perylenequinones within treated tissues and producing organisms is essential. Closely related to hypocrellins, hypomycins are fungal perylenequinones that feature an additional six-membered ring and a less extended conjugated π-system due to the absence of the Δ1(2) double bond. While hypomycins share structural similarities with hypocrellins, their biosynthesis remains unexplored.^14^

We recently reported the redox behavior of these perylenequinones, demonstrating that hypomycins likely originate from the anaerobic reduction of hypocrellins.^15^ This finding helped further refine the absolute configurations of hypomycins C and E. Inspired by reports suggesting that fungi convert these redox-active secondary metabolites (RAMs) into reduced (i.e., nontoxic) forms as a self-protection mechanism,^16–18^ we hypothesized that the intramolecular cyclization (Figure S1) of the hypocrellins to hypomycins in fungi (i.e., *Shiraia* sp.) could be driven by modulating redox homeostasis. By this terminology, we specifically refer to interventions that alter the extracellular microenvironment and, in turn, impact the intracellular balance between oxidative and reductive species, conditions that appear to influence the oxidative cyclization of hypocrellin scaffolds. If confirmed, this would mark a significant advancement in understanding the redox-mediated chemical derivatization and functional roles of perylenequinones within their native organism. Additionally, it would provide insights into the redox stability and fate of these PDT agents under hypoxic (low oxygen) and highly reducing conditions, which are particularly relevant to cancer biology.^19,20^

From a redox perspective, aerobic organisms rely on molecular oxygen as the terminal electron acceptor during oxidative metabolism. Under hypoxic or anaerobic conditions, however, alternative electron acceptors are required to maintain redox homeostasis and cellular viability. In some organisms, such as bacteria, RAMs like phenazines, quinones, and flavins can fulfill this role by reversibly accepting and donating electrons.^21–28^ As Albert Szent-Györgyi famously stated, “Life is nothing but an electron looking for a place to rest”—a sentiment that captures the essence of our investigation into how perylenequinones may mediate fungal redox balance in oxygen-limited environments.

To evaluate whether redox homeostasis could be leveraged to regulate hypomycin bioproduction, we first investigated the impact of chemical reducing agents (i.e., electron donors) on the fermentation and metabolite profiles. For this purpose, glutathione (GSH) with a mild reduction potential (*E*_0_′ = −240 mV for disulfide-thiol GSSG/2GSH redox exchange) was chosen. GSH is naturally abundant, reaching concentrations of up to 10 mM in yeasts and filamentous fungi, where it plays essential roles in metabolism and survival (Figures S2, S3, and Table S1).^29–32^

We found that the reducing effect of GSH facilitated intramolecular cyclization in both *ent*-shiraiachrome A (**1**) and hypocrellin (**2**), significantly enhancing the bioproduction of hypomycins A (**3**), C (**4**), and E (**5**) (Scheme 1 and Figure S4). Notably, hypomycin accumulation correlated with GSH concentration, peaking at the highest tested level without any observable toxicity (Figures 1A, 2A, S4, and Table S2). The absence of GSH-induced toxicity under aerobic growth conditions, where O_2_ is available to mitigate reductive stress, aligns with a recent report on *Saccharomyces cerevisiae*,^33^ a unicellular, nonfilamentous fungus from a different taxonomic class than *Shiraia* sp., though both belong to the phylum *Ascomycota*.

**Scheme 1:**
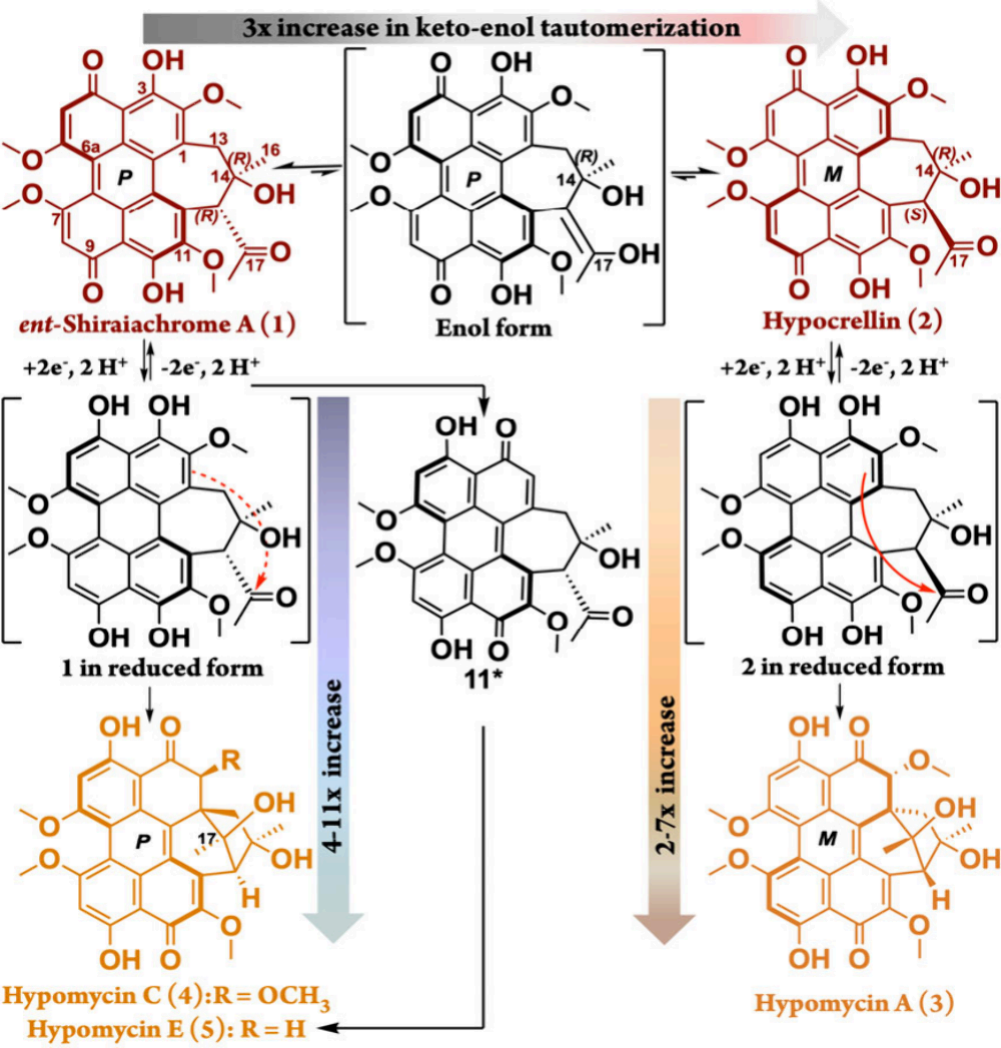
Structural Relationships between Hypocrellins (1-2) and Hypomycins (3-5) Illustrate the Production of the Latter under Reducing and/or Anaerobic Conditions

**Figure 1:**
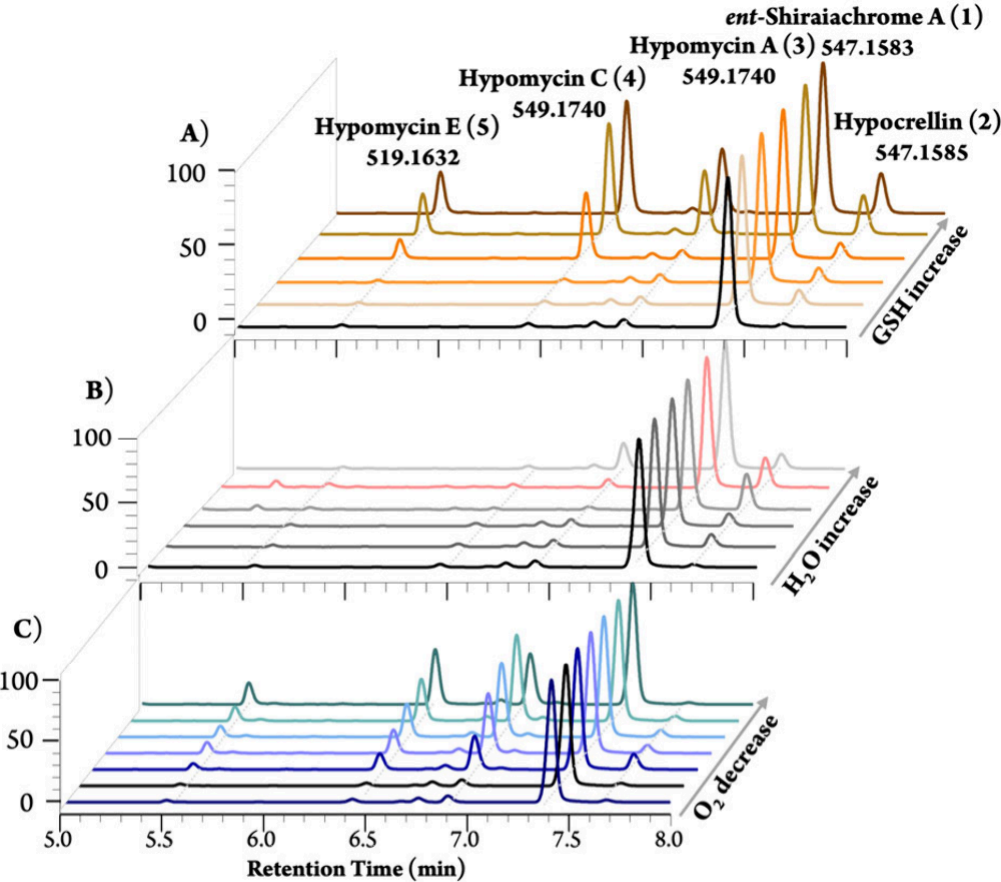
Qualitative comparison of chromatographic data, derived from photodiode array detection across varying A) GSH concentrations (∼4.8 mM–4.8 M), B) moisture contents (0–80%), and C) atmospheric (0–100%) O_2_ levels. Each chromatogram is plotted at 310 nm.

**Figure 2:**
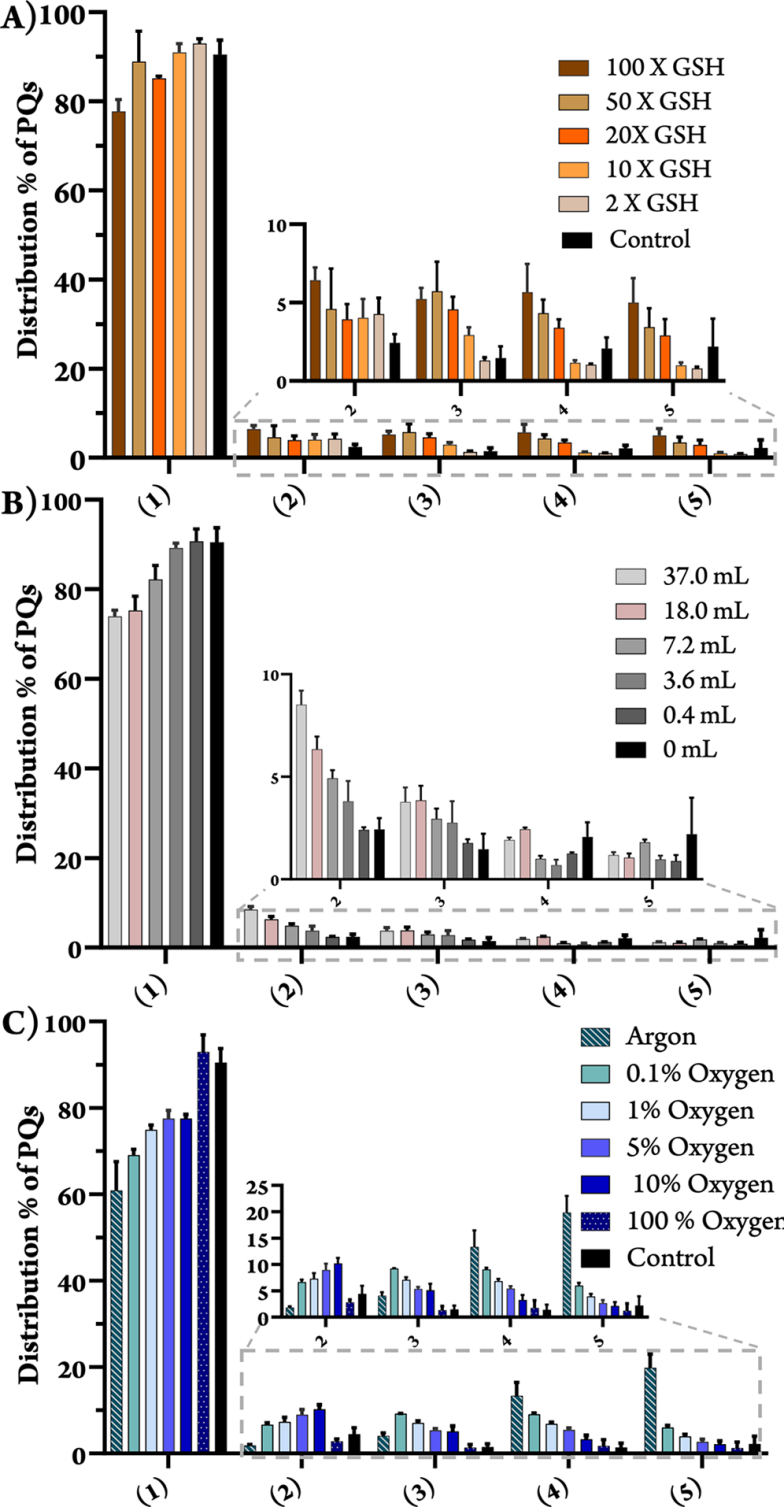
Distribution percentage of perylenequinones per flask A) in different GSH concentrations, B) among controls 1–6 treated with different volumes of nanopure water, and C) in different atmospheric conditions. Values are the mean ± SD of three biological replicates, and the distribution of each compound was calculated as (the amount extracted in mg/total amount of PQs in the flask) × 100%. Statistical significance is shown in Table S4.

Previously, we discovered through chemical reduction^15^ that hypocrellin (**2**) serves as the precursor to hypomycin A (**3**), while *ent*-shiraiachrome A (**1**) is the precursor for both hypomycins C (**4**) and E (**5**) (Scheme 1). Under highly reducing fermentation conditions, we observed a marked decrease in **1** and a corresponding increase in **4** and **5** (up to 7-fold). In contrast, levels of hypocrellin (**2**) remained unchanged or slightly increased, while hypomycin A (**3**) exhibited a 5-fold increase (Figures 1A, 2A).

Since GSH is delivered to the cells in aqueous aliquots, we sought to distinguish the effects of moisture from those of GSH by examining the impact of added water on the metabolic profile. We found that higher moisture content increased the amount of **2** by approximately 3-fold. This result suggests that additional water during fermentation could influence either the atropoisomerization of the common biosynthetic precursor prehypocrellin or the keto–enol tautomerization in both **1** and **2** (Scheme 1). The latter is likely mediated by disrupting their intramolecular hydrogen bonds between the C-17 carbonyl and C-14 hydroxy groups. This disruption shifts the equilibrium initially toward the enol form, followed by a kinetically driven redistribution between the two ketone forms, **1** and **2**. Our calculations predict an activation barrier of 7.4 kcal mol^–1^ for the formation of **1** and 3.5 kcal mol^–1^ for **2**, with **1** being more stable than **2** by ∼ 12.6 kcal mol^–1^ (Figure S5). Ultimately, this process enhances the production of **2** and, consequently, **3** (Figures 1B and 2B).

Notably, we previously observed and reported a similar effect in dimethyl sulfoxide (DMSO),^15^ a potent disruptor of hydrogen bonds. While DMSO is generally considered genetically inactive and is widely used as a solvent in drug-screening assays,^7,8^ its impact on PDT agents such as hypocrellins may induce reactivity and compromise drug integrity.

Overall, increased water content led to a four- to 6-fold increase in hypomycin A (**3**) compared to the dry control, consistent with its close relationship to the production of **2**. In contrast, the overall levels of hypomycins C (**4**) and E (**5**) remained largely unchanged across the various aqueous controls. Results from the first approach demonstrate that the use of a chemical electron donor can be leveraged to enhance intramolecular cyclization, leading to higher production of hypomycins (**3**-**5**). We have also shown that moisture content can disrupt the keto–enol tautomerization governing the axial chirality of hypocrellins, ultimately favoring a kinetic product distribution. The flip in the axial chirality from *P*(*S*) in **1** to *M*(*R*) in **2** results from a configuration change at C-15, shifting from 15*R* in **1** to 15*S* in **2**.

As an orthogonal approach, we then investigated how O_2_ availability affects redox homeostasis and hypomycin bioproduction (Figures S2 and S3). To begin, fungal cultures were incubated under strictly anaerobic (argon) conditions either from day 1 or after a 7-day aerobic growth period to assess differences in metabolic output. Continuous anaerobic growth yielded no perylenequinones, consistent with their O_2_-dependent biosynthesis via flavin-dependent monooxygenases, oxidases, and multicopper oxidases.^34^ However, switching to argon after 7 days of aerobic fermentation restored production of perylenequinones to control levels, although with a markedly different distribution of compounds **1**-**5** (Figure S6). Metabolic profiling under O_2_-free and -limited atmospheres revealed that anaerobic conditions induced intramolecular cyclization (Figure S1), increasing production of hypomycins (**3**-**5**) (Figures 1C and S7). Specifically, the levels of hypomycins C (**4**) and E (**5**) increased approximately nine- to 11-fold when the fungi were grown strictly under argon, while hypomycin A (**3**) exhibited only a 3-fold increase. This difference is likely due to the dry nature of the argon atmosphere suppressing the formation of its precursor, **2**. In oxygen-limited flasks, the decrease in O_2_ availability also directly correlated with elevated production of hypomycins (Figure 2C, Table S3).

Interestingly, the yield of **3** improved substantially, by factors of four to seven, in cultures grown under 0.1%, 1.0%, 5.0%, and 10.0% O_2_. This aligns with the observation that these flasks retained some moisture due to static gas incubation, favoring the production of **2** and thus **3**. In contrast, cultures under 100% dry O_2_ exhibited increased accumulation of **1**, while levels of **2** and **3** decreased, underscoring the influence of both O_2_ and moisture on the redox transformations.

Under oxygen-limited conditions, the profiles of hypomycins C (**4**) and E (**5**) shifted significantly. Compound **4** increased by four- to 6-fold compared to the control, while **5** showed a marked increase only at the lowest oxygen level (i.e., 0.1% O_2_). Strikingly, under argon, **5** became the major hypomycin produced, likely due to the replacement of methoxy groups at C-2 of precursor **1** by hydride ions, followed by intramolecular cyclization (Figures S1 and S8). The absence of moisture (i.e., a proton source) and O_2_ appears to enhance this process, whereas moisture and/or O_2_ suppress hydride availability, thereby reducing the production of **5** relative to that of **4**. The relative abundance of **5** decreased from 32.6% under argon to 8.7%–2.8% across increasing O_2_ concentrations. Importantly, compound **11**, a demethoxylated derivative of **1**, formed via hydride nucleophilic attack and previously known only as an unnatural product from chemical reduction,^15^ was detected here in biological experiments (Figures S1 and S8), specifically under argon and 0.1% O_2_. This observation highlights its likely role as a biosynthetic precursor to hypomycin E (**5**).

Abiotic control experiments conducted under varied redox and atmospheric conditions revealed no changes in compound quantity, relative ratios, or formation of new products for either hypocrellins (**1**-**2**) or hypomycins (**3**-**5**). Experimental details are provided in the Supporting Information (Table S5 and Figures S9–S10). These results confirm the stability of these metabolites under illumination and support that the observed variations in secondary metabolite profiles arise from biologically mediated processes rather than degradation or light-induced reactions.

These findings reveal that low oxygen levels stimulate the intramolecular ring closing (IRC) in a manner similar, though not identical, to the effect of high electron flow (i.e., high concentrations of chemical reductant) observed in the previous experiment. Notably, low O_2_ concentrations or high electron donor concentrations shift the equilibrium to the right in the following equations:

$$1\overset{2\overset{®}{e}}{\underset{O_{2}}{⇄}}1^{2-}\overset{\mathrm{IRC}}{\to }4\;\;\;\;\;\;\;\;2\overset{2\overset{®}{e}}{\underset{O_{2}}{⇄}}2^{2-}\overset{\mathrm{IRC}}{\to }3$$

We previously demonstrated that the rapid intermolecular oxidation of reduced perylenequinones by O_2_ in solution hinders slower intramolecular ring-closing/cyclization.^15^ However, our current findings reveal that, within the organism, under aerobic growth conditions with an excess electron donor, this cyclization in both **1** ^2–^ and **2** ^2–^ competes effectively with their reoxidation on biologically relevant time scales. Notably, based on our previous work,^15^ NMR monitoring of this anaerobic cyclization confirmed the formation of **4** and **3** rather than their doubly reduced counterparts (**4** ^2–^ and **3** ^2–^) as major cyclized products, respectively, reinforcing that this process actively consumes reducing equivalents, effectively funneling electrons from metabolism into irreversible cyclization pathways that yield hypomycins.

To compare the efficiency of atmospheric and chemical redox stimuli on the overall production of perylenequinones by the microorganism, we plotted the levels of *ent*-shiraiachrome A (**1**) against hypomycins C (**4**) and E (**5**), and hypocrellin (**2**) against hypomycin A (**3**) (Figure 3). Perylenequinones production remained sustained under hypoxic and reductive conditions. Fungal viability, confirmed by hyphal growth on fresh nutrient agar and accelerated growth within anaerobic cultures (Figure S11), together with morphological assessments, indicated survival and maintenance of metabolic activity despite prolonged stress. These results imply that these redox-active metabolites may function as alternative electron acceptors in the absence of O_2_ or enhance the rate of consumption of O_2_ by facilitating electron transfer from the cells, thereby promoting anaerobic survival or alleviating reductive stress. Our previous electrochemical studies further demonstrated that the cyclized hypomycins (i.e., **3**-**5**) are approximately 600 mV stronger electron donors than their parent hypocrellins (i.e., **1** and **2**), rendering them more reactive toward O_2_, a reactivity that is constrained under anaerobic conditions.

**Figure 3:**
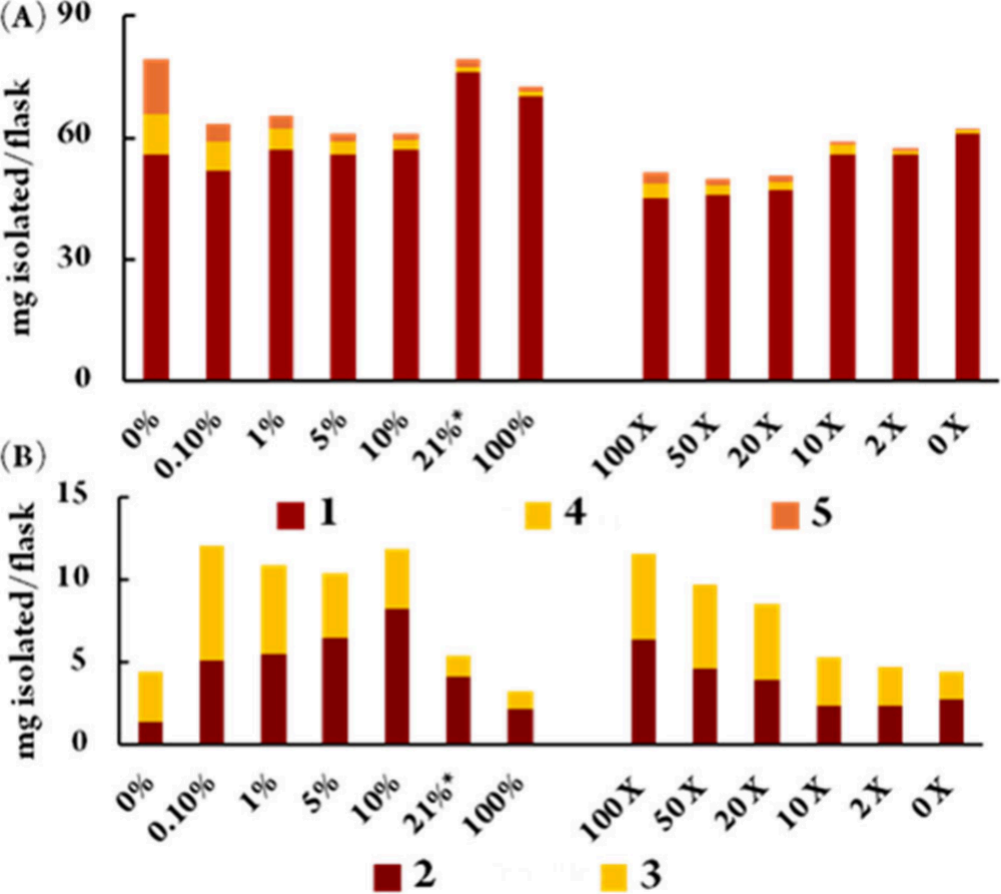
Correlations between hypomycins (**3**-**5**) and their parent compounds, **1** (A) and **2** (B), under varying oxygen levels (left) and reducing agent concentrations (right). *21% represents the natural abundance of dioxygen in air.

Interestingly, the metabolic profile under an argon atmosphere (Figure 3) closely resembles the product distribution observed in a previously reported anaerobic chemical reduction experiment,^15^ where the reduction of **1** resulted in 11% of **5** and 5% of **4**. Similarly, **2** was chemically converted into hypomycin A (**3**) with a 65% yield. Moreover, our energy calculations (Table S6) indicate that among the hypomycins, compound **3** is the most thermodynamically stable, which may explain its higher cyclization efficiency under both chemical and biological conditions. This similarity suggests that analogous reaction pathways, such as the intramolecular ring closing and hydride nucleophilic attack, are present at the cellular level in *Shiraia* sp. during dry hypoxic fermentation, mirroring those observed in nonbiological laboratory settings. Therefore, this biotransformation is likely a nonenzymatic redox-mediated cyclization,^35^ distinct from many known enzymatic cyclization processes.^36–39^ Consistent with this, cell lysate experiments did not yield any detectable cyclization or interconversion of **1** and **2** under the tested conditions (Figures S12–S14), underscoring that these nonenzymatic, redox-mediated transformations require an intact cellular environment. Such molecular derivatization under specific cellular reduction potentials, particularly in hypoxic and reductive cancer microenvironments, could influence the stability and efficacy of PDT agents, such as hypocrellins, by altering their photophysical properties. Understanding these transformations is essential for optimizing drug design and ensuring their effectiveness in biomedical applications.

These findings underscore the intricate role of redox modulation in shaping perylenequinone metabolism, with implications for both fungal survival and PDT agent stability under hypoxic and reductive conditions. The observed transformations suggest that these RAMs, similar to those in bacterial systems,^21–28^ may serve as alternative electron acceptors, helping regulate cellular redox homeostasis in oxygen-limited environments. Moreover, the striking parallels between chemical and biological redox processes highlight opportunities to leverage these insights in metabolic engineering and drug development. Future studies will be crucial to further elucidate the mechanistic implications of these transformations and their broader significance in both natural and therapeutic contexts.

## Supplementary Material



## Notes

Details on experimental procedures, materials and methods, optimization and control studies, computational studies, and additional Supporting Information (PDF)

## ∥

F.S.T.K.: Department of Chemistry and Biochemistry, The Ohio State University, Columbus, Ohio 43210, United States
