# Supplementary material for: Redox Homeostasis as a Key Regulator of Intramolecular Cyclization in Fungal Perylenequinones
Source: ACS Chem Biol. 2025 Aug 12;20(9):2063–8. doi: 10.1021/acschembio.5c00369 (PMC12455569; doi:10.1021/acschembio.5c00369)
Supplement: Supplementary file 1 [file cb5c00369_si_001.pdf]

Supporting Information

Redox Homeostasis as a Key Regulator of Intramolecular Cyclization in Fungal Perylenequinones

Reema A. Al-Qiam,[1] Firoz S. T. Khan,[1] Huzefa A. Raja,[1] Tyler N. Graf,[1] Cedric J. Pearce,[2] Nicholas H. Oberlies,[1,*] and Shabnam Hematian[1,3*]

[1] Department of Chemistry and Biochemistry, University of North Carolina at Greensboro, Greensboro, North Carolina 27402, United States

[2] Mycosynthetix, Inc., Hillsborough, North Carolina 27278, United States

[3] Department of Chemistry, Virginia Tech, Blacksburg, Virginia 24061, United States

Table of Contents:

General Experimental Procedures 3

Figure S1: Proposed mechanism of the intramolecular cyclization process. Compound **11** was isolated previously (Figure S8) and considered as an intermediate to hypomycin E (**5**). The dashed arrows represent a backside attack, while the solid arrows represent an attack from the front. 8

Figure S2: Experimental workflow of the two studies discussed in this paper. A) The effect of glutathione addition on the production of perylenequinones. B) The effect of different atmospheric conditions on the production of perylenequinones. The figure was generated using the Mind the Graph website. 9

Figure S3: Photographs of the various fungal growths. In all cases, these were taken looking down the neck of the Erlenmeyer flask. A) Samples where various concentrations of GSH were added. B) Samples that were grown under different atmospheric conditions. 9

Table S1: Details for the GSH addition experiments. In all treatments and controls, three biological replicates were used. 10

Figure S4: Qualitative comparison of the production of a suite of perylenequinones after the addition of various concentrations of GSH. The values represent the mean of three biological replicates, each of which was analyzed in triplicates ± SD (*$p < 0.05$, **$p < 0.01$ versus the control group). Peak areas were derived from LC-MS measurements, and all samples were analyzed at 0.2 mg/mL. Control peak areas were averaged from the six control groups noted in Table S1. 10

Table S2: Isolated amounts of a suite of perylenequinones (i.e., compounds **1**-**5**) after treating with various concentrations of GSH. Data are presented as mg/flask ± SD (n = 3 biological replicates). 11

Figure S5: A visual representation of the energy changes occurring during chemical tautomerizations and interconversion between **1** and **2**, showing the relative energy levels of both compounds and the enol intermediate. Energies were calculated for all the compounds and the transition states using B3LYP/6-31G* hybrid density functional using H_2O as a medium of solvation.[4] Calculations were performed using Spartan '10 software. Minimized structures based on crystal data were used as starting points. Transition state geometries were predicted using the "Guess Transition State" feature and validated at the same level of theory. The reported values represent relative free energy difference (ΔE) in kcal/mol compared to compound **1**. Transition state structures (TS1 and TS2) and their corresponding imaginary frequencies are shown; the negative frequencies confirm the identity of the transition states. 12

Figure S6: Qualitative comparison of chromatographic data, derived from photodiode array detection in an argon experiment, transferred to an anaerobic environment at different times. Where red showed the chromatogram of the samples grown in an argon environment from day 1. Cyan is the sample grown under Argon after 7 days, and Black is the control (air) 13

Figure S7: Qualitative comparison of the production of a suite of perylenequinones (i.e., compounds **1**-**5**) after culturing under a suite of atmospheric conditions. The values represent the mean of three biological replicates, each of which was analyzed in triplicate ± SD (*$p < 0.05$, **$p < 0.01$ versus the control group). Peak areas were derived from LC-MS measurements, and all samples were analyzed at 0.2 mg/mL. 13

Table S3: Isolated amounts of a suite of perylenequinones after growth under different atmospheres. Data are presented as mg/ flask ± SD (n = 3 biological replicates). 14

Table S4: Significance distribution of all perylenequinones among different conditions. All statistical analyses were conducted using a student t-test with *$p < 0.05$ and ** $p < 0.01$. 15

Figure S8: UPLC-PDA-HRESIMS chromatogram of the analysis of the Argon experiment (visualized by PDA) showing the peak of compound **11** (isolated in previous work) and considered as an intermediate for **5**. [2] The red dots on the structures are there to illustrate how these molecules differ from **1**. 16

Table S5: Abiotic control experiments examining the effect of the environmental and additive effect on pure compounds **1-5** in absence of fungal cells. 17

Figure S9: Qualitative comparison of chromatographic data, derived from photodiode array detection across the abiotic control experiments showing A) Compounds **1** and **2** mixtures used in this experiment. B) Compounds **1** and **2** mixtures maintained under Argon atmosphere and LED for 7 days. C) Compounds **1** and **2** mixtures maintained in high GSH concentration and LED for 7 days. D) Compounds **1** and **2** mixtures maintained in ambient conditions under LED for 7 days. Each chromatogram is plotted at 310 nm. 18

Figure S10: Qualitative comparison of chromatographic data, derived from photodiode array detection across the abiotic control experiments showing A) Compounds **3**-**5** mixtures used in this experiment. B) Compounds **3**-**5** mixtures maintained under Argon atmosphere and LED for 7 days. C) Compounds **3**-**5** mixtures maintained in high GSH concentration and LED for 7 days. D) Compounds **3**-**5** mixtures maintained in ambient conditions under LED for 7 days. Each chromatogram is plotted at 310 nm. 18

Figure S11: Representative photographs of *Shiraia* sp. cultures grown on malt extract agar (MEA) plates under ambient air (control, left) and continuous argon flow (right). The top panels show cultures at 0 hours; the middle panels at 45 hours; and the bottom panels at 135 hours of incubation. For the argon condition, plates were initially purged to remove residual air and then maintained under continuous argon flow in a well-sealed chamber. Anaerobic survival and growth were observed under these conditions. Experiments were performed in triplicate; representative images are shown. 19

Table S6: Calculated relative energies of **1-5**, using B3LYP /6-31G* hybrid density functional using H_2O as a medium of solvation. The structures were all minimized, and the conformer distribution was calculated using the same level of theory, then, the best conformers were selected for energy calculation. 20

Figure S12: ABTS reaction results of native protein reacted with ABTS (Left), native protein (Middle), and denatured protein reacted with ABTS (Right). 20

Figure S13: Schematic representation of the lysate experiments. 21

Figure S14: Protein lysate experiment results showing no evidence of any transformations of **1-5** with incubation with native/ denatured protein in presence or absence of GSH. The peak observed at 6 minutes in samples containing native protein is attributed to a surfactant (PEG) present in the lysis buffer. The plotted chromatogram used PDA data. .... 22 & 23

Table S7: Cartesian coordinates of all the optimized geometries shown in Figure S5. .... 24 & 25

References: 25

Experimental Section

General Experimental Procedures

NMR spectra were recorded in $CDCl_3$ using either a JEOL ECA-500 spectrometer or a JEOL ECS-400 spectrometer, the latter of which was equipped with a high-sensitivity JEOL Royal probe and a 24-slot autosampler. The NMR chemical shifts were referenced to $CDCl_3$ (δ_H 7.26) and were used to confirm sample purity. UPLC-PDA-HRESIMS data were collected via an LTQ-Orbitrap XL mass spectrometry system (Thermo Finnigan, San Jose, CA, USA) connected to a Waters Acquity UPLC system, which used a BEH Shield RP18 column (Waters, 1.7 μm; 50 mm × 2.1 mm) heated to 40 °C. The mobile phase consisted of CH_3CN-H_2O (0.1% formic acid) and used a gradient system that started at 15:85 and increased to 75:25 over 12 min at a flow rate of 0.3 mL/min. MS data were collected from *m/z* 150 to 2000 in the positive mode. All analytical and preparative HPLC experiments were carried out using a Varian Prostar HPLC system equipped with ProStar 210 pumps and a Prostar 335 photodiode array detector (PDA). HPLC data were collected and analyzed using Galaxie Chromatography Workstation software (version 1.9.3.2, Varian Inc.). A LUNA PFP column (Waters, 5 μm; 250 mm × 21.2 mm) was used for preparative HPLC. Flash chromatography was carried out using a Teledyne ISCO CombiFlash Rf 200 that was equipped with both UV and evaporative light-scattering detectors and Silica Gold columns. Deionized H_2O was purified by a PURELAB flex 1 Analytical Ultrapure Water System (ELGA) to obtain nano-pure H_2O with a specific resistance of 18.2 MΩ cm at room temperature. 2,2′-azino-bis(3-ethylbenzothiazoline-6-sulfonic acid) diammonium salt (ABTS) was purchased from Oakwood Chemical with ≥98% purity as determined by HPLC. The energy calculations and transition state geometries were performed using Spartan '10 software. All structures were geometry optimized, and energy minimized using the B3LYP/6-31G* hybrid density functional theory, with water as the solvation medium. Transition state predictions were validated by frequency analysis, and the corresponding Cartesian coordinates are provided in Table S7.

a) <u>Strains, Media, and Culture Conditions</u>

Fungal strain MSX60519 was identified as a *Shiraia* sp. in the family Shiraiaceae, as detailed previously.[1, 2] It was maintained on malt extract agar (MEA; Difco) and transferred regularly to fresh MEA Petri plates. An agar plug

from the leading edge of the MEA culture was transferred to a sterile tube with 10 mL of YESD (20 g soy peptone, 20 g dextrose, 5 g yeast extract, 1 L nanopure H_2O, and autoclaved at 120 °C). The YESD culture was grown for ~10 days on an orbital shaker (100 rpm) at room temperature (~23 °C). This inoculum was then aseptically transferred to solid breakfast oatmeal (Quaker oats) in 250 mL Erlenmeyer flasks, as described previously.[1] Briefly, this medium consisted of 10 g of oatmeal and 17 mL of nanopure H_2O, all of which is autoclaved. The flasks were incubated at room temperature for 15 days under continuous LED light exposure; these conditions were shown previously to enhance the production of both hypocrellins and hypomycins.[1]

To determine the optimal timing for modifying fermentation conditions, three experimental groups were established (all in triplicate). The first group was incubated under anaerobic conditions from day 1. The second group was initially incubated under aerobic conditions and subsequently transferred to an anaerobic atmosphere on day 7. The third group served as the control and was maintained under aerobic conditions for the entire 14-day incubation period (Figure S7). Based on the results of this experiment, we decided to modify the atmospheric conditions or introduce reducing agents after allowing sufficient time for the fungus to initiate secondary metabolism (i.e., after 7 days).

b) <u>Study 1: Enhancing the Reducing Power of *Shiraia sp.* through Glutathione Supplementation as an Electron-Rich Reductant</u>

The fungal cultures were grown and propagated on oatmeal in Erlenmeyer flasks, as described above. The cultures were fermented for the first 7 days under ambient conditions. Then, the reduced form of L-glutathione (Thermo Scientific Chemicals; GSH, Purity 98+%) was added to the flasks in various concentrations starting after 1 week of cultivation. The GSH solution was added portion-wise over 4 -5 days, and each day, the GSH solution was freshly prepared in nano-pure H_2O (0.13 M). The solution was injected into the flasks using a long-needle syringe equipped with a filter (VWR Sterile Syringe Filter, 0.22 µm).

The specific details were Group 1 (2× GSH): 0.1 mL of the GSH solution daily; Group 2 (10× GSH): 0.9 mL of the GSH solution daily; Group 3 (20× GSH): 1.8 mL of the GSH solution daily; Group 4: (50× GSH): 1.8 mL of the GSH solution twice per day; Group 5: (100× GSH): 3.7 mL of the GSH solution twice per day. In groups 1-3, the solution was added once a day for 4 days, while for the high concentrations (groups 4 and 5), the same procedure was applied, but it was implemented twice a day, for 5 days. The control flasks were treated with nano-pure H_2O in various volumes in three biological replicates that corresponded to the volume added in each group [control 1: 0.1 mL, control 2: 0.9 mL, control 3: 1.8 mL, control 4:1.8 mL (twice a day), and control 5: 3.7 mL (twice a day)].

Validation of the Experimental Design: The fungus (strain MSX60519) was grown in various concentrations of GSH, ranging from 15 mg to 1.5 g. These values were chosen as a multiplication of the natural content of GSH in fungi[3] (10 mM under normal conditions, see Table S1). The cultures were grown in triplicate in each group in a

lightbox over oatmeal for seven days. Then, the GSH treatments were started on day 8 in different portions every 12 hours (Groups 4 and 5; due to the solubility limit of GSH in H_2O) or every day (Groups 1-3). We also tested the effect of neat H_2Oadded by treating five controls with the corresponding amount of H_2Oas the five groups, along with one dry control. The first step was qualitatively comparing the different conditions as measured via HRESIMS peak areas (Figure S4). Additionally, the total amount of perylenequinones were isolated and compared among the five groups and six control gropes (Table S2), where on average each flask produced 60.4 ±4.4 mg of compounds **1**-**5** with no significant differences between all of them.

c) <u>Study 2: Enhancing Hypomycin Production via Controlled Fermentation Atmospheres to Promote Hypocrellin Conversion</u>

The fungal growths were separated into six groups, each of which consisted of three biological replicates. These cultures were cultivated under normal conditions in a lightbox for seven days to allow sufficient time for the biosynthesis of metabolites. Then, on the eighth day, the flasks underwent purging with different gases. For Group 1 (argon group), the flasks were fermented under a rigorously anaerobic environment by maintaining them on a Schlenk line under dry argon for 7 days. For Groups 2-5 (limited oxygen), the flask headspace was purged with argon for 15 min, followed by the injection of 0.3, 3.0, 15.0, or 30.0 mL of dry oxygen using a three-way, long syringe needle, establishing approximately 0.1%, 1%, 5%, and 10% oxygen content in the flask atmosphere, respectively. For Group 6 (pure oxygen group), the flasks were purged with dry oxygen for 15 min and connected to an oxygen-filled balloon to maintain positive pressure for 7 days. For Group 7 (Control), the flasks were sealed and fermented under ambient air. Ultra-high purity grade argon (99.9999%) and oxygen (99.994%) gases were purchased from Airgas. Both gases were further dried by passing through a short column of supported P_4O_{10} (Aquasorb, Mallinckrodt). All the flasks were maintained under these atmospheric conditions for 7 days before extraction, following the procedures detailed below.

Validation of the Experimental Design: We examined the effect of environmental oxygen on the rate of intramolecular cyclization, as noted by production of hypomycins (i.e., compounds **3**-**5**). To do so, we compared the metabolic profile of *Shiraia* fungus fermented in different oxygen-containing environments ranging from an oxygen-free environment to a 100% oxygen air environment, along with the control flasks grown under normal air. To ensure that the fungi will handle the new environments, we let the fungus grow for the first seven days in normal conditions (under light), so that biosynthesis of secondary metabolites was established, and then we applied the new atmospheres. Based on the total amount of perylenequinones produced under each condition (Table S3), we believe that the various atmospheric conditions did not interrupt the biosynthesis of perylenequinones (with an average of total perylenequinones produced ~ 78.3 ± 8.6 mg across all experiments). Instead, it was the distribution of each perylenequinone (i.e., compounds **1**-**5**) that was altered.

d) <u>Abiotic Control Experiments</u>

In these experiments, we evaluated whether the experimental factors such as atmospheric conditions, redox additives, or light exposure, could impact the distribution of perylenequinones. We tested the effects of light exposure in air, oxygen-limited conditions, and in the presence of ~20× GSH solution to determine whether any side reaction, such as decomposition, intramolecular cyclization, or enol-keto tautomerization could occur abiotically. Our results confirmed that these transformations occur only in the presence of fungal cells.

To investigate this, we designed six experimental conditions, each performed in duplicate (Table S5). In the first three conditions, a mixture of **1** (40.0 mg) and **2** (4.0 mg) was dissolved in a minimal amount of acetonitrile (~2 mL) and added to oatmeal media (identical to the standard fermentation setup but without fungal mycelium):

A) Two flasks were purged with argon, sealed, and incubated under continuous light for 7 days.

B) Two flasks were treated with four daily doses (1.8 mL each) of freshly prepared GSH solution (0.13 M in nanopure H_2O), added dropwise over 4 days to mimic the 20× GSH condition. These were also kept under continuous light for 7 days.

C) Two flasks were maintained under ambient air and continuous light for the same period.

In parallel, three corresponding abiotic control experiments were conducted using a mixture of hypomycins **3-5** (3 mg each). After 7 days, all flasks were extracted, and the metabolic profiles were analyzed by LC/MS (Figures S9 and S10). These experiments revealed no changes in compound ratios, quantities, or product formation, confirming that both hypocrellins and hypomycins remain stable under light exposure across all tested conditions. These findings further support that the observed variations in secondary metabolite profiles are not the result of degradation pathways or light-induced reactions but instead arise from biologically mediated processes.

e) <u>Cell-Lysate Experiments</u>

Protein extracts from *Shiraia* sp. were prepared using the **Minute™ Total Protein Extraction Kit for Microbes with Thick Cell Walls** (Invent Biotechnologies, Inc., Catalog No. YT-015). To begin, fungal cells were harvested by centrifugation at ~4000 × *g* for 15 minutes at 4 C in a 25 mL centrifuge tube. The resulting pellet (~1.5 mL) was washed with 1 mL nanopure water and centrifuged again under the same conditions for 10 minutes. After removing the supernatant completely, ~80–90 mg of protein extraction powder was added directly to the pellet. For protein solubilization, ~100 μL of native buffer (for the active protein group) or denaturing buffer (for the denatured control) was added. The cell mixture was manually ground for ~5 minutes using the provided pestle with twisting force. An additional ~50 μL of extraction buffer was then added, followed by vortexing to enhance cell rupture.

Lysates were centrifuged at 4000 × *g* for 15 minutes at 4 °C, and the resulting supernatant was collected and concentrated using 10 kDa spin filters to obtain the crude protein mixture used in the following assays.

To verify enzymatic activity, the protein extracts (native and denatured) were incubated with ABTS (7.0 mM). A color change to green (indicative of ABTS oxidation) was observed in the native protein sample but not in the denatured control (Figure S12).

For metabolite stability assays, compounds **1-5** were prepared as 40 mg/mL stock solutions (2 mg in 50 μL MeCN), and ~2.0 μL (~0.8 mg/mL final concentration) was added to each reaction tube. All samples (Figure S13) were incubated at room temperature for 6 hours, then extracted with ethyl acetate. The organic layer was collected, dried, and reconstituted in MeOH for LC/MS analysis (Figure S14).

f) Extraction and Fractionation of the Fungal Cultures

Extraction of the solid cultures was performed as described previously.[1] Briefly, each culture was chopped and shaken overnight in 90 mL of 1:1 CH_3OH–$CHCl_3$. The slurry was vacuum filtered, and 90 mL of $CHCl_3$ and 100 mL of DI H_2O were added to the filtrate. This mixture was stirred for 30 min before being transferred into a separatory funnel. The bottom layer was drawn off, evaporated to dryness, and then reconstituted in 100 mL of 1:1 CH_3OH–CH_3CN and 100 mL of hexanes. The biphasic solution was shaken vigorously and transferred into a separatory funnel. The CH_3OH/CH_3CN layer was drawn off and evaporated *in vaccuo*.[1]

g) Quantification and Purification of Perylenequinones

Extracts of the various fungal growths were analyzed via UPLC-PDA-HRESIMS to compare the chemical profiles of the cultures with each other. A small aliquot was prepared using HPLC grade MeOH: dioxane (1:1) from all the extracts at the same concentration (i.e., 0.2 mg/mL), and the LC-MS data were collected in triplicate for qualitative comparison. Afterward, each sample was dissolved in $CHCl_3$, adsorbed onto Celite 545, and subdivided into three fractions via normal-phase flash chromatography using a gradient solvent system of hexanes–$CHCl_3$–MeOH at an 18 mL/min flow rate and 50 column volumes over 29 mins. The second fraction was concentrated in perylenequinones. The second fraction of each extract (i.e., the perylenequinone-rich fraction), which ranged in amount from ~ 80-150 mg/flask, was subjected to HPLC purification over a LUNA-PFP preparative column using a mobile phase consisting of CH_3CN-H_2O (0.1% formic acid) and using a gradient system that started at 30:70 and increased to 60:40 over 30 min at a flow rate of 20.0 mL/min to yield compounds **1**-**5**.

h) Statistical Analysis

All experiments were carried out with three biological replicates. Student's t-test was used as a significance test to compare the mean values of each group vs controls. All results are expressed as mean ± standard deviation (SD). The level of significance was set at $^{*}p < 0.05$ and $^{**}p < 0.01$ (Table S4).

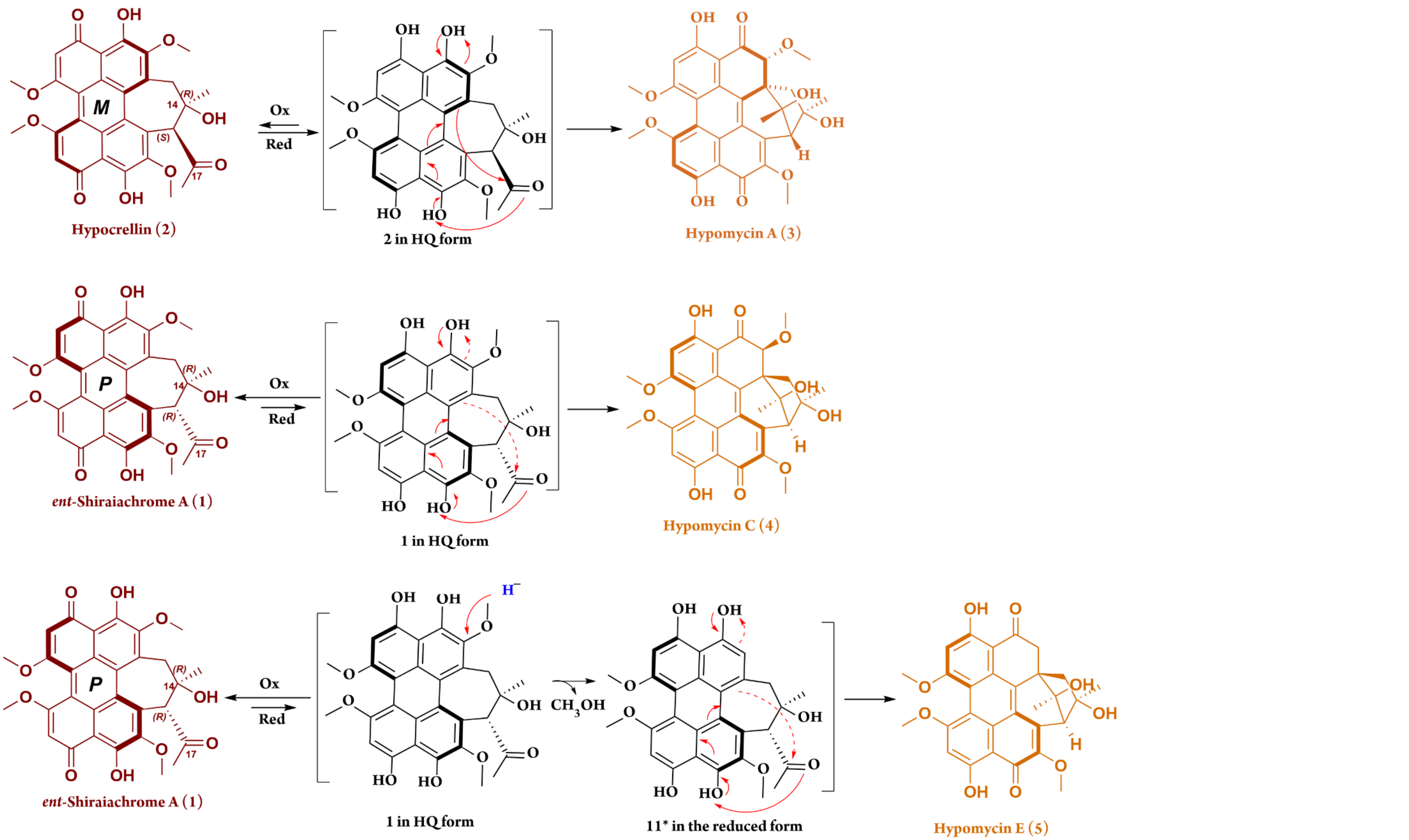

Figure S1: Proposed mechanism of the intramolecular cyclization process. Compound **11** was isolated previously (Figure S8) and considered as an intermediate to hypomycin E (**5**). The dashed arrows represent a backside attack, while the solid arrows represent an attack from the front.

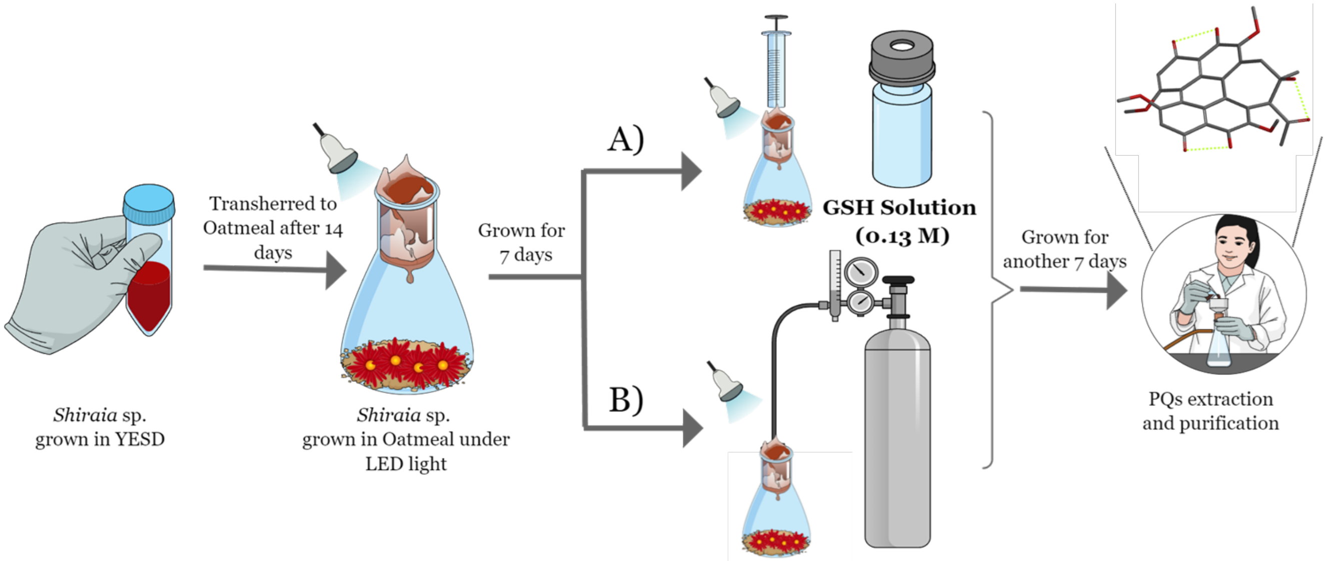

Figure S2: Experimental workflow of the two studies discussed in this paper. A) The effect of glutathione addition on the production of perylenequinones. B) The effect of different atmospheric conditions on the production of perylenequinones. The figure was generated using the Mind the Graph website.

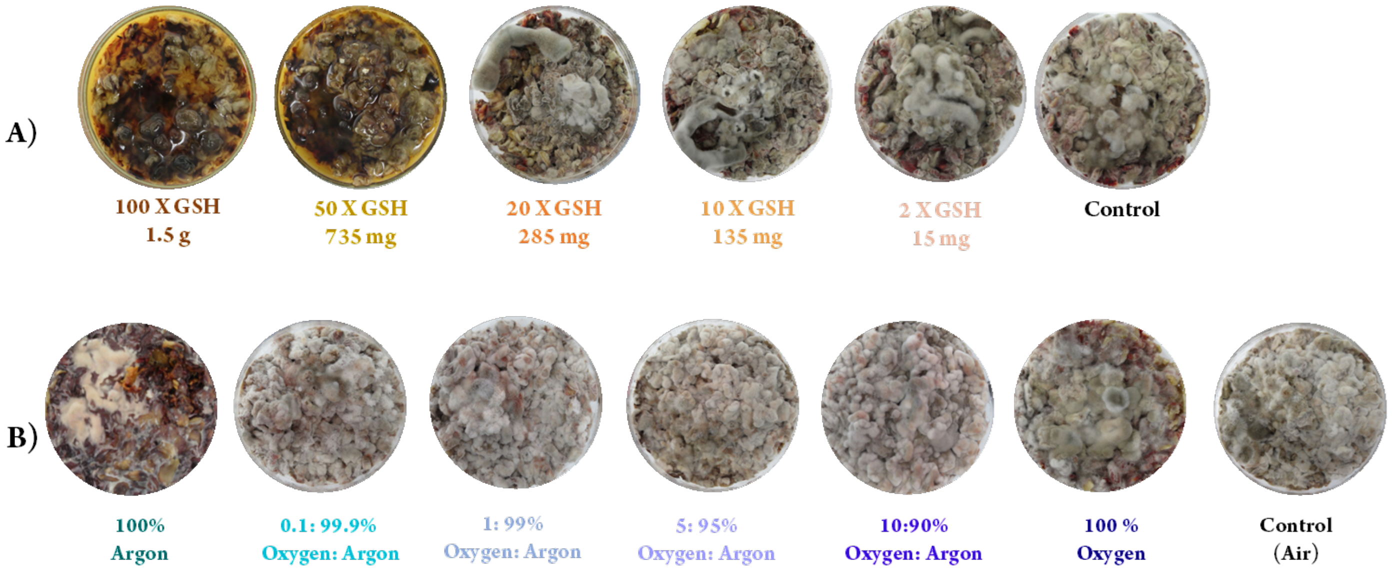

Figure S3: Photographs of the various fungal growths. In all cases, these were taken looking down the neck of the Erlenmeyer flask. A) Samples where various concentrations of GSH were added. B) Samples that were grown under different atmospheric conditions.

Table S1: Details for the GSH addition experiments. In all treatments and controls, three biological replicates were used.

**Group name** (amount. GSH added)	**2X** (15 mg)	**10X** (135 mg)	**20X** (285 mg)	**50X** (735 mg)	**100X** (1.5 g)	**Control***
**Total volume of Stock (0.13M)**	0.4 mL	3.6 mL	7.2 mL	18.0 mL	37.0 mL	-
**Treatment /day**	0.1 mL/day for 4 days	0.9 mL/day for 4 days	1.8 mL/day for 4 days	1.8 mL twice a day for 5 days	3.7 mL twice a day for 5 days	See below
**Details about the control groups:**<br>* A series of controls were also created for each of Groups 1-5, and these were treated with an equivalent amount of nano-pure water in three biological replicates, mirroring the treatment given to experimental groups. Alternatively, the dry control in this column was simply grown without the addition of any extra nano-pure H_2O.						
Control name	Control-1	Control-2	Control-3	Control-4	Control-5	Dry control
Volume of nano-pure H_2O added	0.4 mL	3.6 mL	7.2 mL	18.0 mL	37.0 mL	0.0 mL

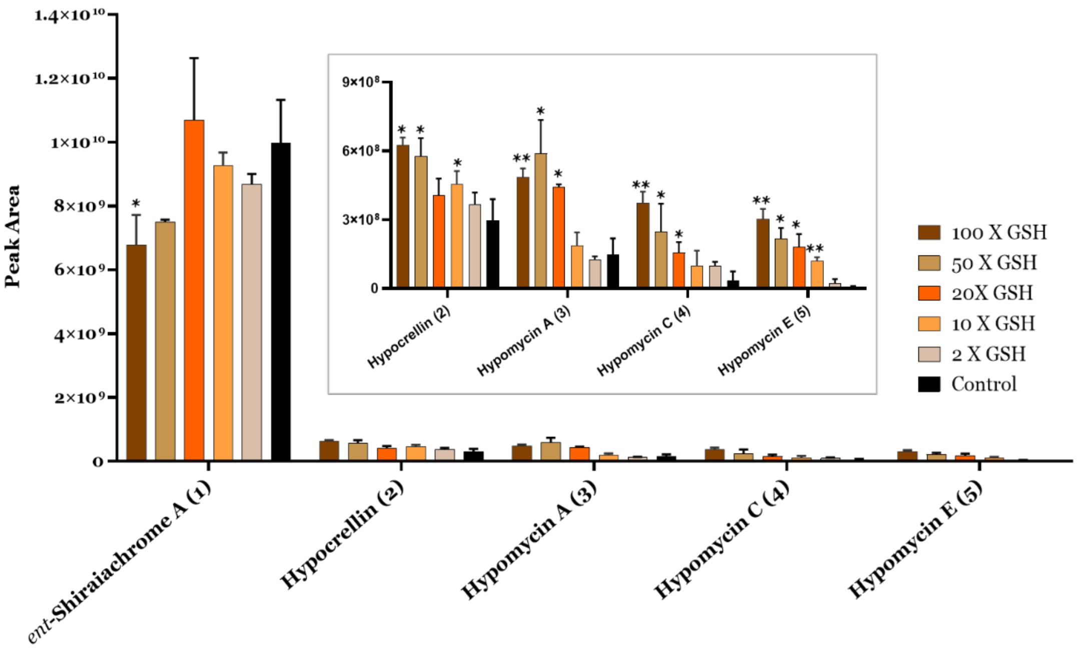

Figure S4: Qualitative comparison of the production of a suite of perylenequinones after the addition of various concentrations of GSH. The values represent the mean of three biological replicates, each of which was analyzed in triplicates ± SD (*$p < 0.05$, **$p < 0.01$ versus the control group). Peak areas were derived from LC-MS measurements, and all samples were analyzed at 0.2 mg/mL. Control peak areas were averaged from the six control groups noted in Table S1.

Table S2: Isolated amounts of a suite of perylenequinones (i.e., compounds **1**-**5**) after treating with various concentrations of GSH. Data are presented as mg/flask ± SD (n = 3 biological replicates).

**Condition** (Amount added)	***ent*-Shiraiachrome A (1)**	**Hypocrellin (2)**	**Hypomycin A (3)**	**Hypomycin C (4)**	**Hypomycin E (5)**	**Total PQs**
100 X GSH (1.5 g)	44.1± 2.8	3.6 ± 2.8	2.9± 0.3	3.2± 1.0	2.8± 0.9	56.7± 1.8
50 X GSH (735 mg)	46.5± 1.8	2.4± 1.4	2.9± 0.27	2.2± 0.3	1.8± 0.5	55.9± 2.8
20 X GSH (285 mg)	47.4± 4.4	2.2± 0.7	2.5± 0.5	1.9± 0.3	1.6± 0.3	55.6± 5.0
10 X GSH (135 mg)	55.9± 4.9	2.4± 0.6	1.8± 0.2	0.8± 0.0	0.9± 0.3	61.4± 4.2
2X GSH (15 mg)	55.9± 4.9	2.4± 0.6	0.9± 0.1	0.7± 0.1	0.6± 0.1	66.7± 5.4
Control*	61.8 ± 5.2	2.8± 0.7	1.6± 0.1	0.7± 0.0	0.5± 0.1	66.4± 5.5

* Isolated amounts from the control group are the average of the six control groups described in Table S1.

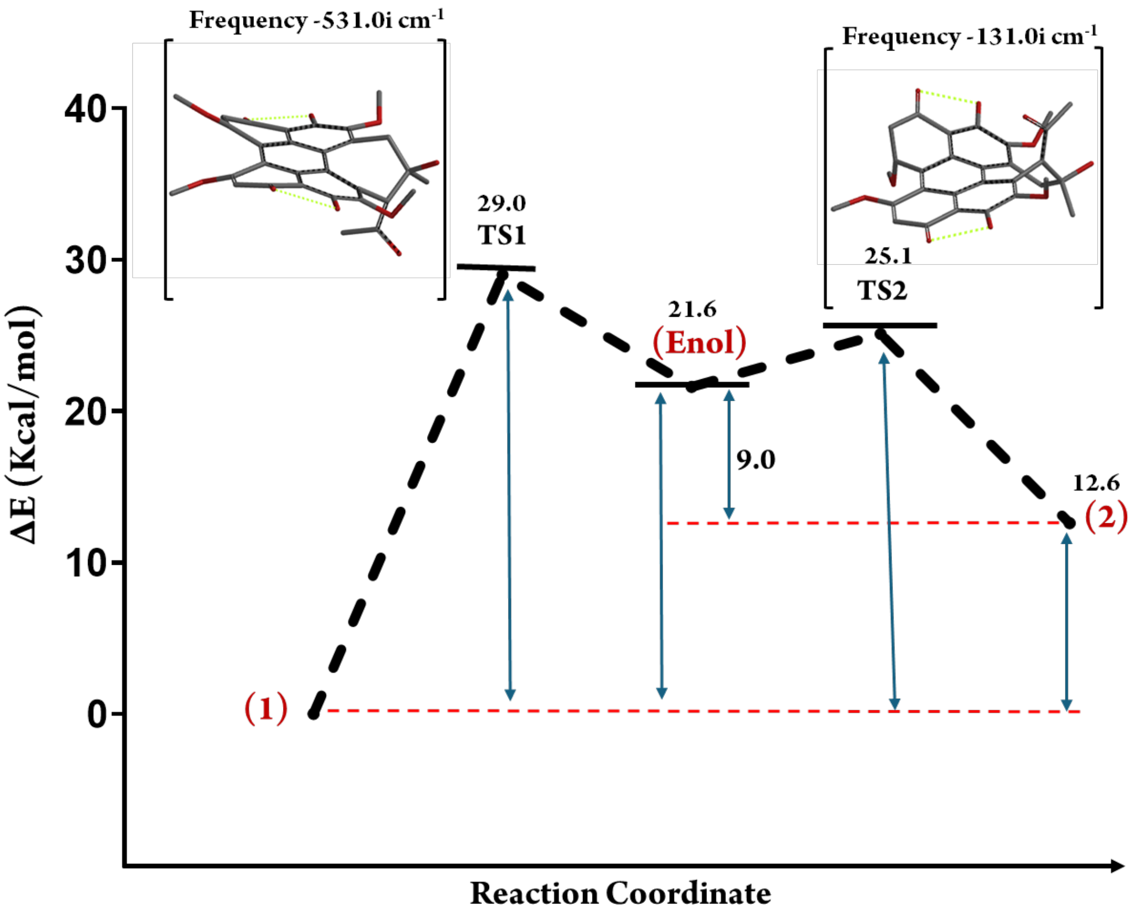

Figure S5: A visual representation of the energy changes occurring during chemical tautomerizations and interconversion between **1** and **2**, showing the relative energy levels of both compounds and the enol intermediate. Energies were calculated for all the compounds and the transition states using B3LYP/6-31G* hybrid density functional using H_2O as a medium of solvation.[4] Calculations were performed using Spartan '10 software. Minimized structures based on crystal data were used as starting points. Transition state geometries were predicted using the "Guess Transition State" feature and validated at the same level of theory. The reported values represent relative free energy difference (ΔE) in kcal/mol compared to compound **1**. Transition state structures (TS1 and TS2) and their corresponding imaginary frequencies are shown; the negative frequencies confirm the identity of the transition states.

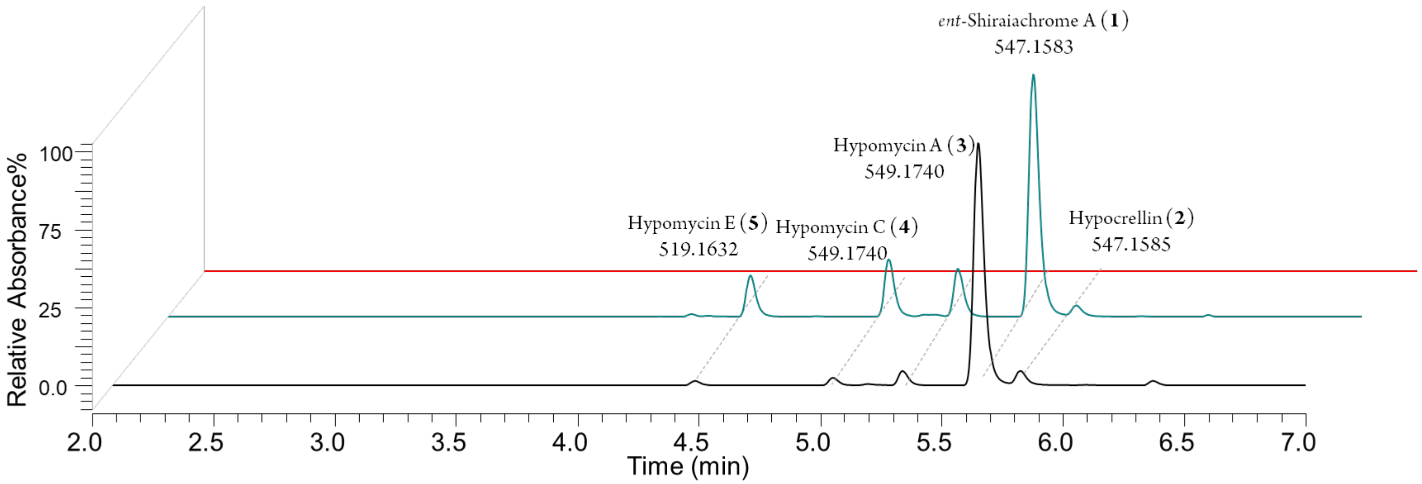

Figure S6: Qualitative comparison of chromatographic data, derived from photodiode array detection in an argon experiment, transferred to an anaerobic environment at different times. Where red showed the chromatogram of the samples grown in an argon environment from day 1. Cyan is the sample grown under Argon after 7 days, and Black is the control (air)

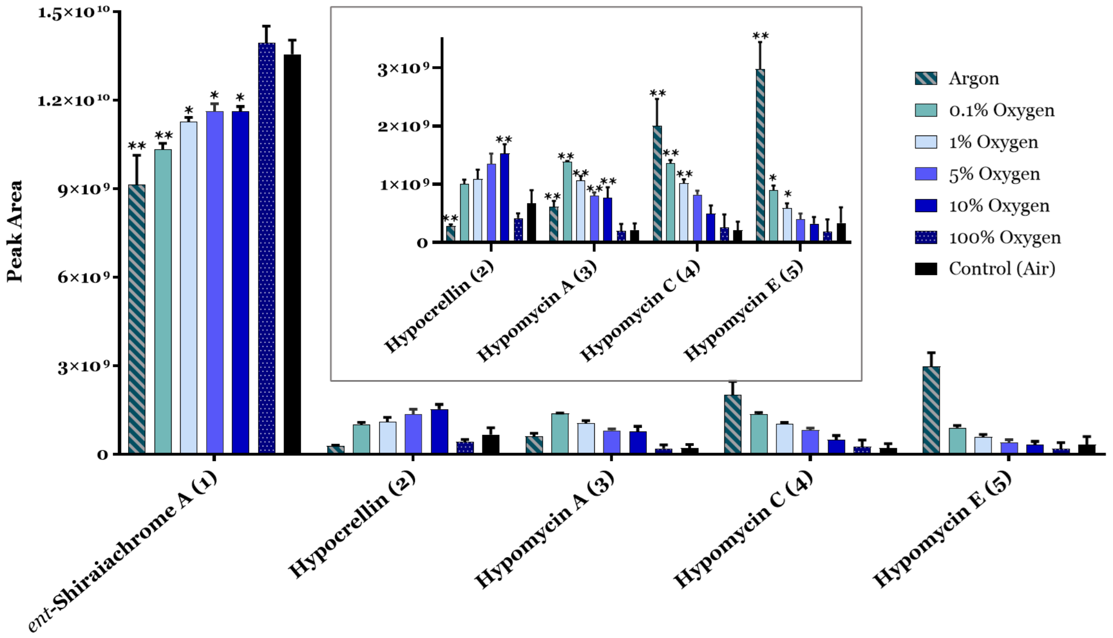

Figure S7: Qualitative comparison of the production of a suite of perylenequinones (i.e., compounds **1**-**5**) after culturing under a suite of atmospheric conditions. The values represent the mean of three biological replicates, each of which was analyzed in triplicate ± SD (**p* < 0.05, ***p* < 0.01 versus the control group). Peak areas were derived from LC-MS measurements, and all samples were analyzed at 0.2 mg/mL.

Table S3: Isolated amounts of a suite of perylenequinones after growth under different atmospheres. Data are presented as mg/ flask ± SD (n = 3 biological replicates).

Condition	*ent*-Shiraiachrome A (1)	Hypocrellin (2)	Hypomycin A (3)	Hypomycin C (4)	Hypomycin E (5)	Total PQs
**Argon**	45.9± 3.5	1.4± 0.9	3.0± 0.2	9.7± 1.0	13.6± 1.5	74.6± 11.5
**0.1:99.9% Oxygen:Argon**	52.3± 3.3	5.1± 0.7	7.0± 1.0	6.9± 0.2	4.5± 0.5	75.8± 4.5
**1:99% Oxygen:Argon**	56.9± 5.9	5.5± 2.1	5.4± 1.0	5.2± 1.0	3.0± 0.5	75.9± 2.8
**5:95% Oxygen:Argon**	56.0± 3.0	6.5± 1.0	3.9± 0.5	2.9± 0.7	1.9± 0.1	72.2± 1.7
**10:90% Oxygen:Argon**	57.3± 3.0	8.2± 1.5	3.7± 1.1	2.4± 0.1	1.6± 0.3	73.9± 3.1
**100% Oxygen**	70.8± 2.9	2.2± 0.5	1.0± 0.4	1.4± 0.4	1.2± 0.5	76.4± 7.9
**Control (air ~21% O_2)**	76.2± 8.3	4.1 ± 0.6	1.3± 0.1	1.3± 0.8	2.1± 1.8	87.9± 5.5

Table S4: Significance distribution of all perylenequinones among different conditions. All statistical analyses were conducted using a student t-test with *$p < 0.05$ and ** $p < 0.01$.

Condition	*ent*-Shiraiachrome A (1)	Hypocrellin (2)	Hypomycin A (3)	Hypomycin C (4)	Hypomycin E (5)
Argon	**	*	**	**	**
0.1:99.9% Oxygen:Argon			**	**	*
1:99% Oxygen:Argon	**		**	**	
5:95% Oxygen:Argon	**	*	**	**	
10:90% Oxygen:Argon	**	*	*		
100% Oxygen	**				
100 X GSH (1.5 g)	**		**	**	**
50 X GSH (735 mg)	*	*	**	**	*
20 X GSH (285 mg)			*	*	*
10 X GSH (135 mg)					
2X GSH (15 mg)	**				

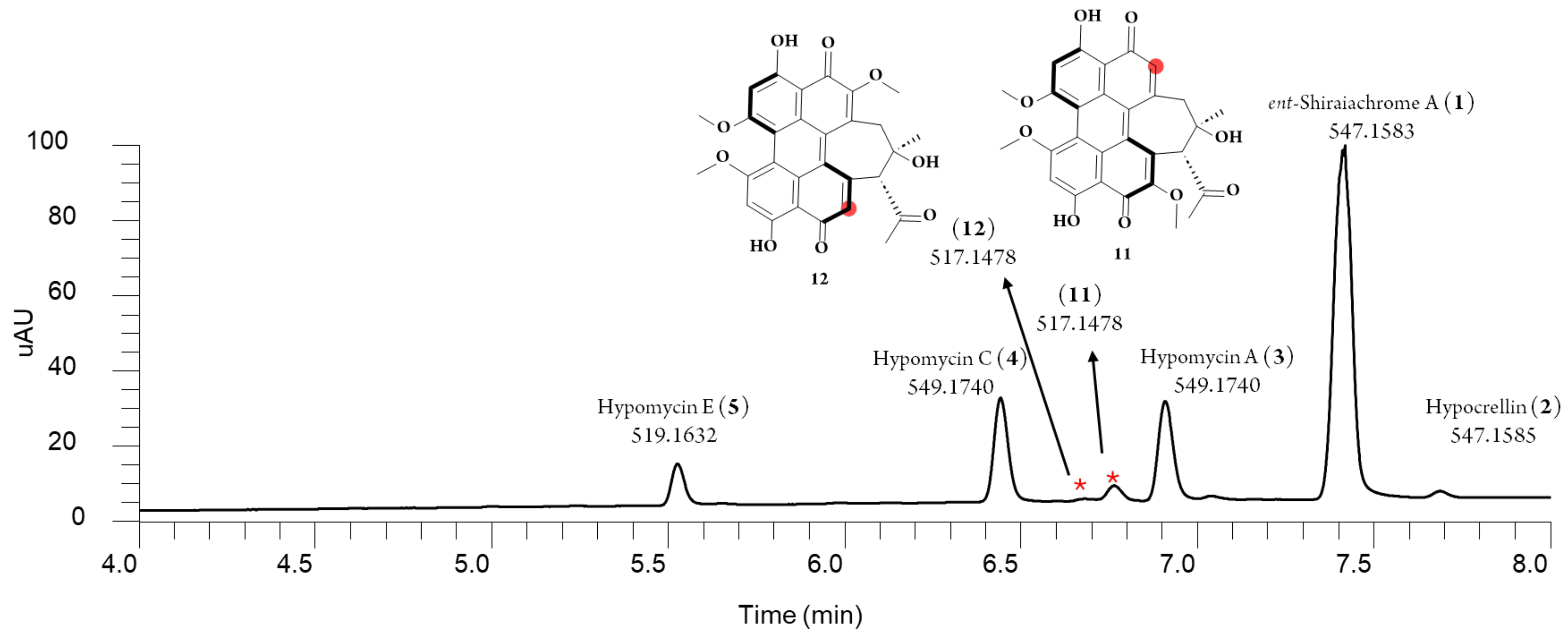

Figure S8: UPLC-PDA-HRESIMS chromatogram of the analysis of the Argon experiment (visualized by PDA) showing the peak of compound **11** (isolated in previous work) and considered as an intermediate for **5**.[2] The red dots on the structures are there to illustrate how these molecules differ from **1**.

Table S5: Abiotic control experiments examining the effect of the environmental and additive effect on pure compounds **1-5** in absence of fungal cells.

Experiment	Conditions[a,b]	Flasks	Conclusions
Hypocrellins under Argon environment	Compounds **1** and **2** maintained for 7 days under oxygen free environment under continuous LED light exposure	2	We observed no changes in the metabolic profile. The overall quantities of compounds remained unchanged, the ratio of compounds **1**:**2** was consistent across all three experiments, and none of the hypomycins (i.e., **3**-**5**) were produced.
Hypocrellins in high GSH conc.	Compounds **1** and **2** maintained for 7 days with 20× GSH solution under continuous LED light exposure	2	
Hypocrellins under ambient atmosphere	Compounds **1** and **2** maintained for 7 days under continuous LED light exposure	2	
Hypomycins under Argon environment	Compounds **3**-**5** maintained for 7 days under oxygen free environment under continuous LED light exposure	2	The experiments suggested the stability of **3**-**5** under a range of conditions. Also, there was no evidence of conversion between the hypomycins.
Hypomycins in high GSH conc.	Compounds **3**-**5** maintained for 7 days with 20×GSH solution under continuous LED light exposure	2	
Hypomycins under ambient atmosphere	Compounds **3**-**5** maintained for 7 days under continuous LED light exposure	2	

[a] In all abiotic experiments, the compounds were incubated with oatmeal to represent the growth media used in the other experiments.

[b] A goal for these experiments was to mimic the concentration of the compounds observed in the fungal extracts. In the first three experiments, a 10:1 ratio of *ent*-shiraiachrome A (**1**):hypocrellin (**2**) was used. In the second set of experiments for compounds **3**-**5**, a 1:1:1 ration of hypomycin A (**3**):hypomycin C (**4**):hypomycin E (**5**) was used.

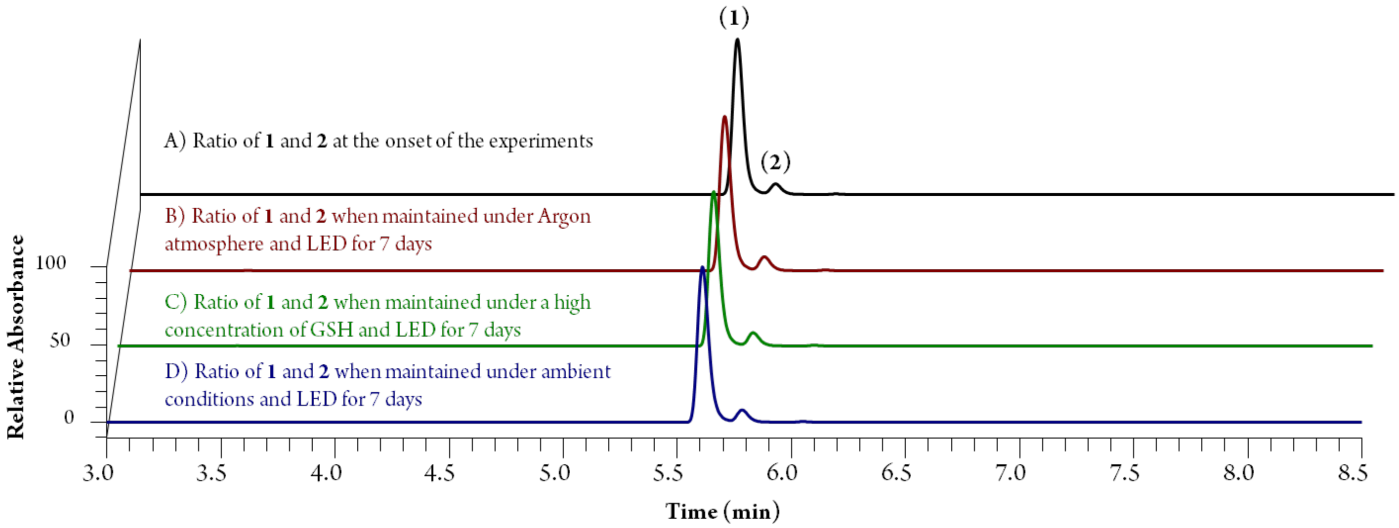

Figure S9: Qualitative comparison of chromatographic data, derived from photodiode array detection across the abiotic control experiments showing A) Compounds **1** and **2** mixtures used in this experiment. B) Compounds **1** and **2** mixtures maintained under Argon atmosphere and LED for 7 days. C) Compounds **1** and **2** mixtures maintained in high GSH concentration and LED for 7 days. D) Compounds **1** and **2** mixtures maintained in ambient conditions under LED for 7 days. Each chromatogram is plotted at 310 nm.

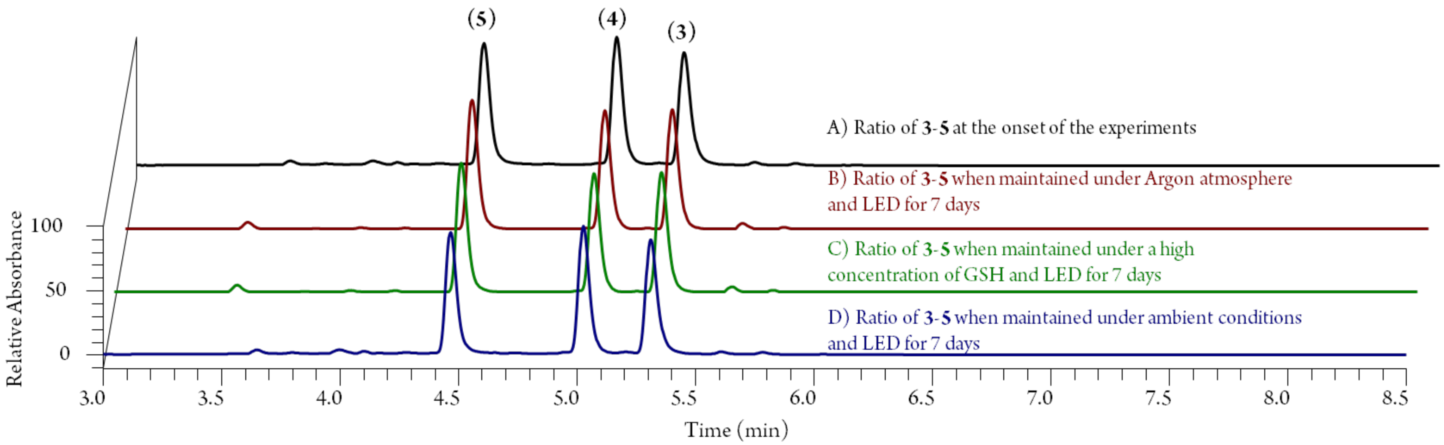

Figure S10: Qualitative comparison of chromatographic data, derived from photodiode array detection across the abiotic control experiments showing A) Compounds **3-5** mixtures used in this experiment. B) Compounds **3-5** mixtures maintained under Argon atmosphere and LED for 7 days. C) Compounds **3-5** mixtures maintained in high GSH concentration and LED for 7 days. D) Compounds **3-5** mixtures maintained in ambient conditions under LED for 7 days. Each chromatogram is plotted at 310 nm.

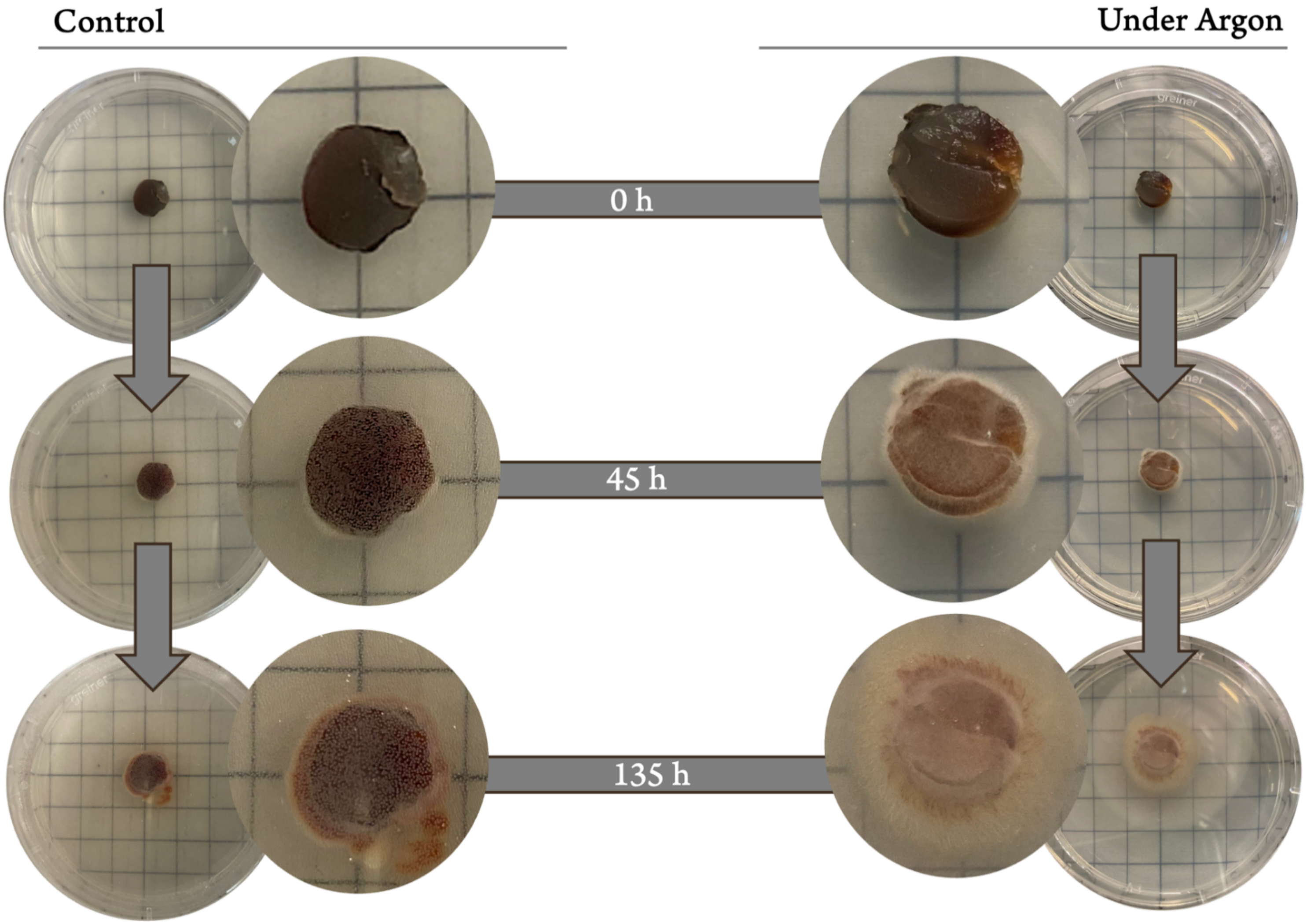

Figure S11: Representative photographs of *Shiraia* sp. cultures grown on malt extract agar (MEA) plates under ambient air (control, left) and continuous argon flow (right). The top panels show cultures at 0 hours; the middle panels at 45 hours; and the bottom panels at 135 hours of incubation. For the argon condition, plates were initially purged to remove residual air and then maintained under continuous argon flow in a well-sealed chamber. Anaerobic survival and growth were observed under these conditions. Experiments were performed in triplicate; representative images are shown.

Table S6: Calculated relative energies of **1**-**5**, using B3LYP /6-31G* hybrid density functional using H_2O as a medium of solvation. The structures were all minimized, and the conformer distribution was calculated using the same level of theory, then, the best conformers were selected for energy calculation.

**Compound**	**ΔE relative to 1(kcal/mol)**	**Dihedral Angle b/w 2 planes (°)**
ent-Shiraiachrome A (**1**)	0	27.70
Hypocrellin (**2**)	+12.6	28.20
Hypomycin A (**3**)	+14.0	23.14
Hypomycin C (**4**)	+26.3	17.13
Hypomycin E (**5**)	+24.6	17.02

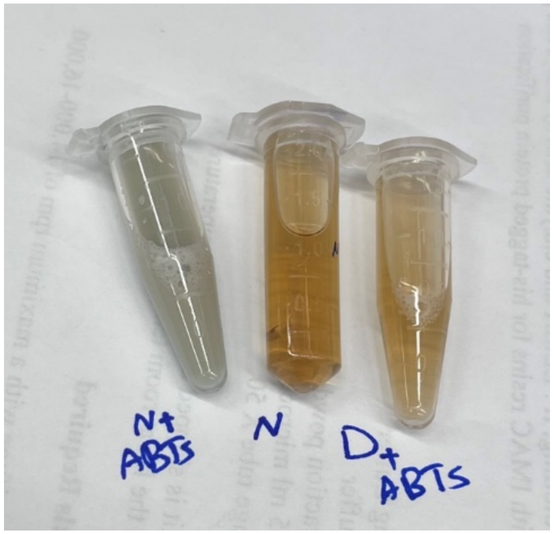

Figure S12: ABTS reaction results of native protein reacted with ABTS (Left), native protein (Middle), and denatured protein reacted with ABTS (Right).

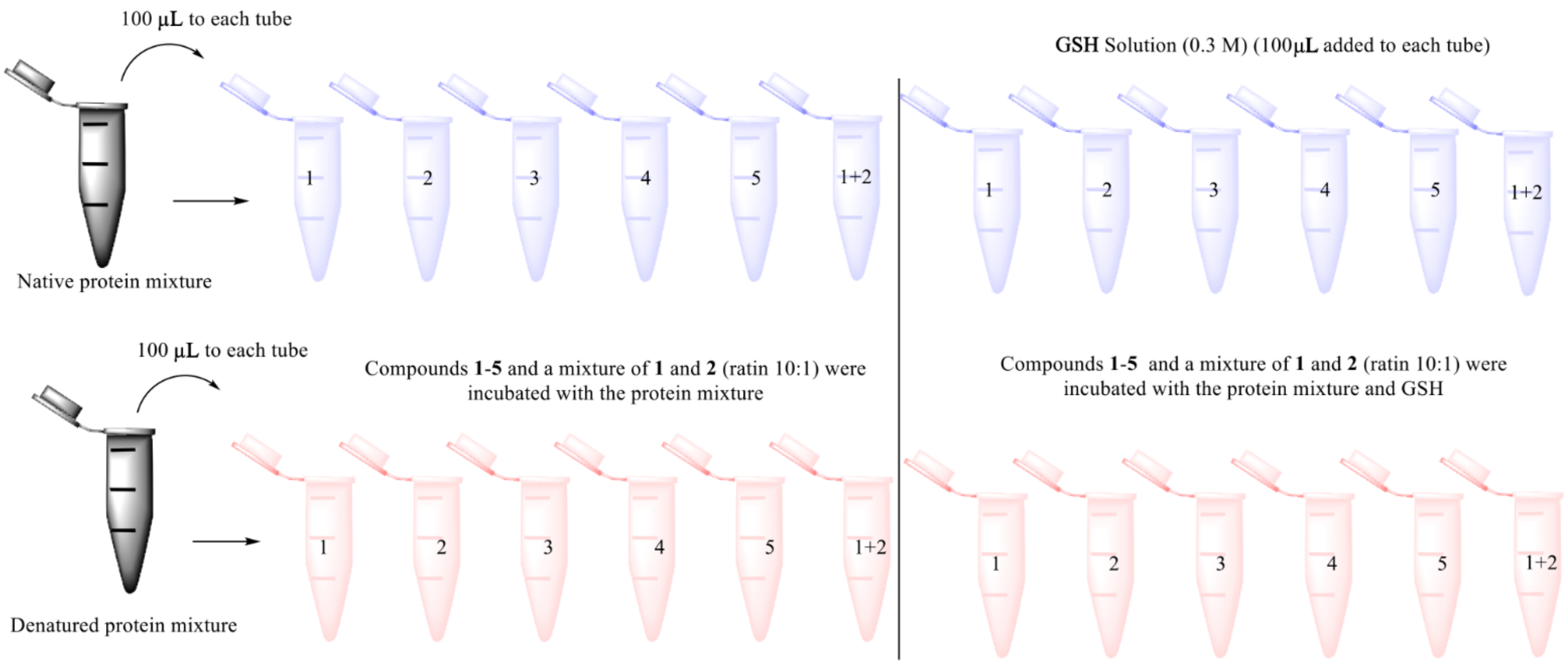

Figure S13: Schematic representation of the lysate experiments.

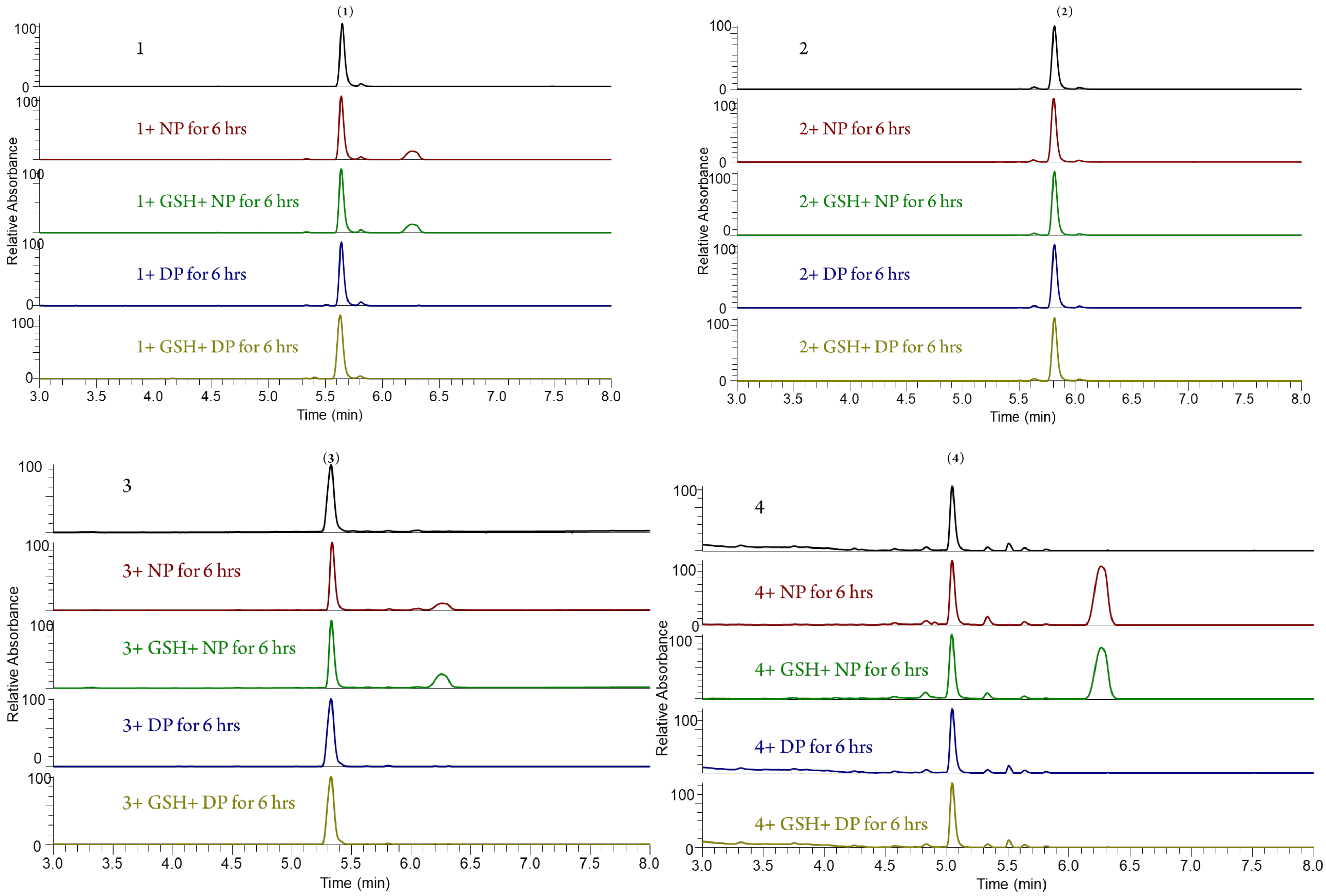
(1)
1
1+ NP for 6 hrs
1+ GSH+ NP for 6 hrs
1+ DP for 6 hrs
1+ GSH+ DP for 6 hrs
Relative Absorbance
Time (min)
(2)
2
2+ NP for 6 hrs
2+ GSH+ NP for 6 hrs
2+ DP for 6 hrs
2+ GSH+ DP for 6 hrs
Relative Absorbance
Time (min)
(3)
3
3+ NP for 6 hrs
3+ GSH+ NP for 6 hrs
3+ DP for 6 hrs
3+ GSH+ DP for 6 hrs
Relative Absorbance
Time (min)
(4)
4
4+ NP for 6 hrs
4+ GSH+ NP for 6 hrs
4+ DP for 6 hrs
4+ GSH+ DP for 6 hrs
Relative Absorbance
Time (min)

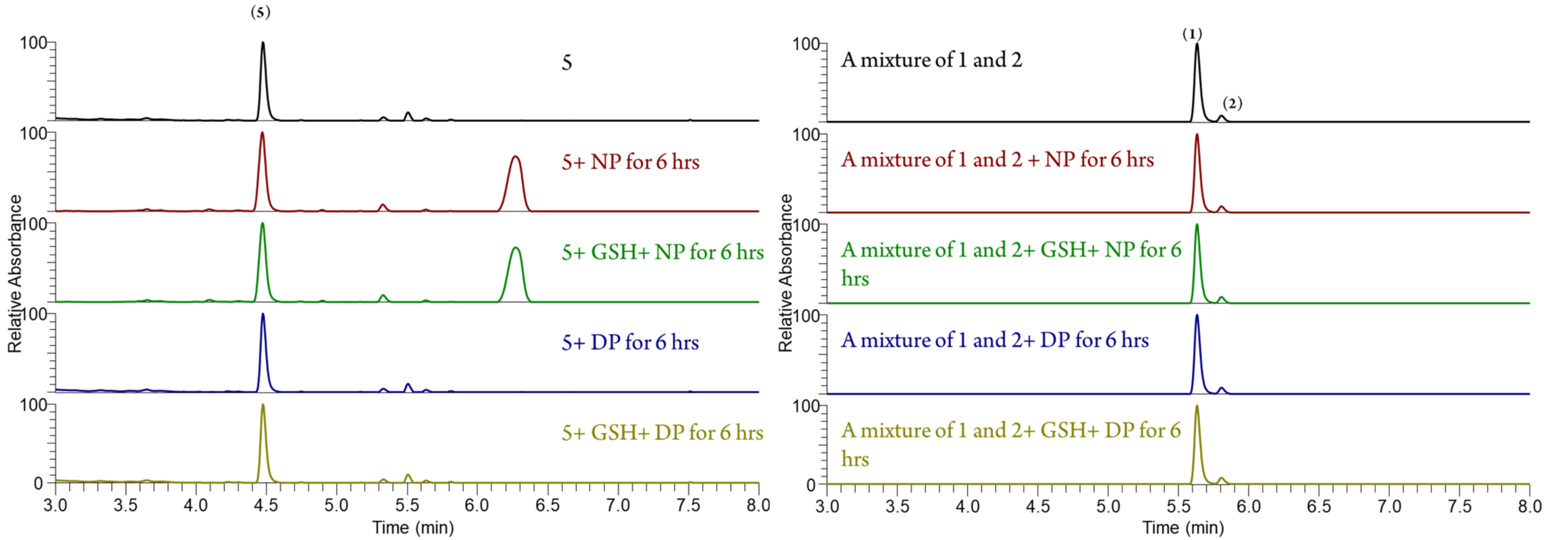

Figure S14: Protein lysate experiment results showing no evidence of any transformations of **1**-**5** with incubation with native/ denatured protein in presence or absence of GSH. The peak observed at 6 minutes in samples containing native protein is attributed to a surfactant (PEG) present in the lysis buffer. The Photo Diode Array data was used in those chromatograms.

Table S7: Cartesian coordinates of all the optimized geometries shown in Figure S5.

(**1**)

Atom	X	Y	Z
1 C C0	2.7360615	-1.1298096	0.5246314
2 C C1	1.6928138	-0.2976154	0.0177588
3 C C2	0.4742909	-0.1481946	0.7272826
4 C C3	0.4222180	-0.6351272	2.0683799
5 C C4	1.4995057	-1.3208126	2.6154987
6 C C5	2.6429118	-1.6384258	1.8189013
7 C C6	3.9022562	-1.4462279	-0.3215065
8 C C7	4.1625691	-0.5407114	-1.4298472
9 C C8	3.2312133	0.3648503	-1.8129537
10 C C9	1.8833118	0.3948969	-1.2313286
11 C C10	0.8485107	1.1163647	-1.7840358
12 C C11	-0.3280970	1.3900878	-0.9977552
13 C C12	-0.6180585	0.5922839	0.1365136
14 C C13	0.8457900	1.6605729	-3.1451029
15 C C14	0.1309748	2.7645933	-3.4625560
16 C C15	-0.7459107	3.3810052	-2.4694637
17 C C16	-1.1462914	2.5018904	-1.3548426
18 C C17	-2.3091834	2.7349323	-0.6283803
19 C C18	-2.7603323	1.7311865	0.2875383
20 C C19	-1.9701371	0.6292076	0.6216316
21 C C20	-0.7711688	-0.4147207	2.9267479
22 C C21	-2.7032284	-0.4605820	1.3457208
23 C C22	-1.9314940	-1.2669925	2.4070347
24 O O23	4.6538603	-2.4103562	-0.0814237
25 O O24	3.5587780	-2.4395637	2.4303355
26 O O25	1.4651534	-1.6026350	3.9683547
27 C C26	1.7560833	-2.9542525	4.3321030
28 O O27	3.4572929	1.3705411	-2.7181561
29 C C28	4.7742955	1.4586462	-3.2599761
30 O O29	-1.1796056	4.5389478	-2.5980122
31 O O30	1.5958811	1.0764180	-4.1538063
32 C C31	1.3215850	-0.3111151	-4.3602791
33 O O32	-3.1149330	3.8247886	-0.7408887
34 O O33	-4.0727862	1.7701647	0.7314002
35 C C34	-4.3877235	2.8841180	1.5727057
36 C C35	-3.4328291	-1.4041613	0.4020420
37 C C36	-3.1417382	-1.3220035	-1.0574587
38 O O37	-4.2576669	-2.2010266	0.8674999
39 O O38	-2.7918776	-1.4654735	3.5294572
40 C C39	-1.4541681	-2.6230234	1.8970726
41 H H1	5.1519408	-0.6209297	-1.9022106
42 H H2	0.1637105	3.2170587	-4.4641654
43 H H3	-0.5639912	-0.7024823	3.9949953
44 H H4	-1.0613307	0.6691117	2.9322133
45 H H5	-3.5321965	0.0543424	1.9404491
46 H H6	4.3239289	-2.5989130	1.8506871
47 H H7	2.8611092	-3.0682529	4.4589433
48 H H8	1.2261668	-3.0831745	5.3099071
49 H H9	1.3740543	-3.6753112	3.5722188
50 H H10	4.7264687	2.3859394	-3.8857834
51 H H11	5.5298103	1.5637944	-2.4442295
52 H H12	4.9933885	0.5645517	-3.8920321
53 H H13	1.9014087	-0.5539990	-5.2855866
54 H H14	1.6886464	-0.9112602	-3.4924078
55 H H15	0.2301952	-0.4793728	-4.5219695
56 H H16	-2.7533531	4.4534842	-1.3927818
57 H H17	-5.3331728	2.5643198	2.0792944
58 H H18	-3.5796833	3.0668691	2.3191474
59 H H19	-4.5574662	3.7924352	0.9445567
60 H H20	-3.5093552	-0.3407670	-1.4500136
61 H H21	-2.0396657	-1.3868022	-1.2322399
62 H H22	-3.6529277	-2.1478008	-1.6096754
63 H H23	-3.5271681	-2.0171789	3.2260083
64 H H24	-0.9709129	-2.5160106	0.8973160
65 H H25	-0.7140120	-3.0511453	2.6171433
66 H H26	-2.3137200	-3.3311722	1.8093784

[**Enol**]

Atom	X	Y	Z
1 C C0	-1.3920345	-2.6592499	-0.0502722
2 C C1	-1.1350293	-1.2723219	0.0157639
3 C C2	0.1813968	-0.8372902	0.2800175
4 C C3	1.1428673	-1.8102292	0.6965991
5 C C4	0.8718304	-3.1905542	0.6050089
6 C C5	-0.3913550	-3.6134021	0.1965388
7 C C6	-2.7643949	-3.1080137	-0.3674580
8 C C7	-3.7474464	-2.0967256	-0.7822973
9 C C8	-3.4614605	-0.7882658	-0.7229541
10 C C9	-2.1714241	-0.3207948	-0.1336877
11 C C10	-1.9204907	1.0240296	0.2343016
12 C C11	-0.5851377	1.4843456	0.2475801
13 C C12	0.4710685	0.5546262	0.1512629
14 C C13	-2.9862988	1.9830483	0.6501882
15 C C14	-2.7414997	3.3010750	0.6802125
16 C C15	-1.3960039	3.8280985	0.4088266
17 C C16	-0.2794088	2.8648379	0.2972854
18 C C17	1.0409114	3.3407992	0.1966680
19 C C18	2.0802244	2.4381022	-0.0068162
20 C C19	1.7711542	1.0716400	-0.0995637
21 C C20	2.4159592	-1.3450773	1.4111518
22 C C21	2.8516236	0.1329377	-0.6298978
23 C C22	3.4947722	-0.8399503	0.4487637
24 O O23	-3.0688439	-4.2967664	-0.3422778
25 O O24	-0.6349340	-4.9561935	0.0556321
26 O O25	1.9312740	-4.0168774	0.8785891
27 O O26	-4.2300846	0.2102911	-1.2860069
28 C C27	-5.4850148	-0.1369974	-1.8583192
29 O O28	-1.2021455	5.0373277	0.3336383
30 O O29	-4.1491625	1.3881683	1.0950043
31 C C30	-5.2327576	2.2209284	1.4889044
32 O O31	1.3219524	4.6835435	0.2179049
33 O O32	3.3882300	2.7770058	-0.2336906
34 C C33	3.9140829	3.7973062	0.6088888
35 C C34	3.1774252	-0.0548065	-2.0363312
36 C C35	2.2680472	0.8780383	-2.8112658
37 O O36	4.4007694	-0.1932378	-2.3933957
38 O O37	4.1051956	-1.9676660	-0.1781841
39 C C38	4.5896286	-0.0963510	1.2107287
40 C C39	1.6288568	-5.2321171	1.5570259
41 H H1	-4.6612344	-2.4985141	-1.1985782
42 H H2	-3.4702118	4.0457391	0.9705884
43 H H3	2.1283896	-0.5607456	2.1246651
44 H H4	2.8198543	-2.1498792	2.0380475
45 H H5	-1.5881917	-5.0927336	-0.1445128
46 H H6	-5.9587865	0.7834560	-2.2120828
47 H H7	-5.3522278	-0.8000671	-2.7190873
48 H H8	-6.1445562	-0.5896209	-1.1108221
49 H H9	-6.0699848	1.5737132	1.7663748
50 H H10	-4.9662058	2.8213883	2.3644640
51 H H11	-5.5590274	2.8573291	0.6599467
52 H H12	0.4805945	5.1932622	0.2485210
53 H H13	3.4812644	3.7768013	1.6153799
54 H H14	4.9894355	3.6197946	0.7096934
55 H H15	3.7868192	4.7786375	0.1430503
56 H H16	1.2110367	0.6883187	-2.6128083
57 H H17	2.5305102	1.9254603	-2.6523877
58 H H18	2.4245524	0.6721836	-3.8760114
59 H H19	4.9624541	-0.4737120	-1.6477204
60 H H20	3.4037812	-2.6057773	-0.4027505
61 H H21	4.1877451	0.7579112	1.7636568
62 H H22	5.0983670	-0.7620237	1.9165890
63 H H23	5.3531573	0.2814853	0.5229554
64 H H24	0.8130729	-5.1174258	2.2793140
65 H H25	1.4186021	-6.0289760	0.8379747
66 H H26	2.5222314	-5.5267638	2.1167116

(**2**)

Atom	X	Y	Z
1 C C0	-1.4882518	0.2958483	-3.6877664
2 O O1	-2.0941526	-1.9308813	-4.0701329
3 O O2	-1.9467183	-3.5690339	-1.6993615
4 C C3	-2.3922503	-2.5202363	0.4028235
5 C C4	-2.1326104	-2.5214416	-1.0675790
6 C C5	-5.1664685	0.4785100	-1.0929314
7 O O6	-3.9669915	0.9625127	-1.7028481
8 O O7	-3.6861973	3.0833895	0.0107138
9 C C8	0.6903602	1.9037509	5.3616659
10 O O9	0.6725301	1.1035435	4.1822902
11 O O10	-2.4970007	4.0675520	2.2425196
12 C C11	3.6371148	2.6980756	3.0148891
13 O O12	2.7119672	1.6453917	3.2801176
14 O O13	2.2253939	-2.9197536	-2.9231791
15 O O14	4.1306628	-2.8662303	-0.9345102
16 O O15	5.0198133	-1.9153096	1.0248842
17 C C16	-1.4439577	-1.1116148	-3.0963347
18 C C17	-2.2172735	-1.1857016	-1.7741816
19 C C18	-0.0176744	-1.5956571	-2.9169171
20 C C19	-1.8611632	-0.0143507	-0.9266130
21 C C20	-2.8744394	0.9867102	-0.8374677
22 C C21	-2.7483146	2.0786945	0.0017144
23 C C22	-1.6858720	2.1113342	0.9475816
24 C C23	-1.6945527	2.9690607	2.0902023
25 C C24	-0.9096867	2.6696449	3.1950022
26 C C25	0.0411098	1.6360736	3.0851556
27 C C26	-0.6529781	0.1622351	-0.2565053
28 C C27	-0.6503809	1.1432783	0.8098500
29 C C28	0.3293067	1.0586034	1.8356186
30 C C29	1.5704219	0.3739021	1.5646618
31 C C30	2.7205174	0.6453104	2.3132497
32 C C31	3.9017475	-0.1122670	2.1737841
33 C C32	3.9358791	-1.0937499	1.2046487
34 C C33	2.9924667	-2.1140046	-0.8329250
35 C C34	2.0206578	-2.0957410	-1.8136951
36 C C35	0.7912725	-1.3678805	-1.6797476
37 C C36	0.5679009	-0.6172283	-0.5206727
38 C C37	1.6440354	-0.5240162	0.4511674
39 C C38	2.8474847	-1.2603784	0.2946210
40 C C39	3.0626068	-2.3239297	-3.9184216
41 H H1	-2.5262264	0.7033788	-3.6241843
42 H H2	-0.7940177	0.9630544	-3.1256135
43 H H3	-1.1820641	0.2564462	-4.7606695
44 H H4	-2.0851965	-2.8419565	-3.7445492
45 H H5	-2.5592778	-3.5597465	0.7757731
46 H H6	-1.5100917	-2.0814114	0.9341653
47 H H7	-3.2905551	-1.8960001	0.6315558
48 H H8	-5.0158332	-0.5480510	-0.6804001
49 H H9	-5.4916511	1.1746198	-0.2814163
50 H H10	-5.9045552	0.4664759	-1.9332157
51 H H11	-4.2099941	3.0099432	-0.8051342
52 H H12	1.3608616	1.3275059	6.0488815
53 H H13	1.1193220	2.9118986	5.1472734
54 H H14	-0.3379674	1.9873797	5.7901499
55 H H15	-2.9514598	4.2729593	1.4136408
56 H H16	3.5719399	3.3416144	3.9286345
57 H H17	4.6733344	2.3033416	2.8844850
58 H H18	3.3191412	3.2630541	2.1084200
59 H H19	4.0021082	-3.4958638	-1.6657089
60 H H20	5.6811766	-1.6838375	1.6953096
61 H H21	-3.3152748	-1.1154402	-2.0639643
62 H H22	-0.0482492	-2.7219881	-3.0625612
63 H H23	0.5823465	-1.1811493	-3.7792697
64 H H24	-1.0296557	3.2479838	4.1205346
65 H H25	4.7549754	0.0766968	2.8385959
66 H H26	4.0924971	-2.1686240	-3.5109383
67 H H27	3.0665645	-3.0759359	-4.7466960
68 H H28	2.6415137	-1.3503634	-4.2684693

(**TS1**)

	Atom		X	Y	Z
1	C	C0	-0.6365997	-1.3279182	-2.4657610
2	C	C1	-0.4947327	-1.1437493	-1.0508523
3	C	C2	-0.4423424	0.1654189	-0.5217639
4	C	C3	-0.8971676	1.2540463	-1.3562370
5	C	C4	-0.9872148	1.0870621	-2.7260612
6	C	C5	-0.7841592	-0.2304363	-3.3076784
7	C	C6	-0.6193508	-2.6860120	-3.0460057
8	C	C7	-0.7526692	-3.7949161	-2.1292152
9	C	C8	-0.7399184	-3.5852414	-0.7850851
10	C	C9	-0.3728658	-2.2797707	-0.1768738
11	C	C10	0.0062860	-2.0962860	1.1421598
12	C	C11	0.0847076	-0.7584330	1.6835409
13	C	C12	-0.0036370	0.3668992	0.8283489
14	C	C13	0.3939861	-3.1806101	2.0672299
15	C	C14	0.4558573	-3.0337644	3.4066115
16	C	C15	0.2446362	-1.7212391	4.0155847
17	C	C16	0.2189168	-0.5756928	3.0979296
18	C	C17	0.3127299	0.7153747	3.6233987
19	C	C18	0.5107209	1.8248488	2.7135063
20	C	C19	0.4075105	1.6603539	1.3466272
21	C	C20	-1.4006314	2.5054736	-0.7157394
22	C	C21	0.8588190	2.8150406	0.4928953
23	C	C22	-0.2907754	3.4868188	-0.3179542
24	O	O23	-0.5698239	-2.8230959	-4.2783441
25	O	O24	-0.7977104	-0.3330359	-4.6413404
26	O	O25	-1.2953151	2.2263872	-3.4273731
27	C	C26	-1.6701338	2.0932689	-4.8250315
28	O	O27	-1.1448098	-4.4712113	0.1539157
29	C	C28	-1.5681230	-5.7968585	-0.2767135
30	O	O29	0.1746642	-1.5465143	5.2361343
31	O	O30	0.7185404	-4.4190296	1.5177789
32	C	C31	2.1147783	-4.5232889	1.1276946
33	O	O32	0.2785084	0.9916112	4.9255288
34	O	O33	0.9718299	3.0264091	3.2293156
35	C	C34	-0.0773579	3.8185429	3.8600059
36	C	C35	2.1253898	2.5441374	-0.3290999
37	C	C36	2.5931020	1.1436071	-0.5610501
38	O	O37	2.7623713	3.4895694	-0.7422639
39	O	O38	-0.9748416	4.3503107	0.6274416
40	C	C39	0.1907064	4.3187812	-1.4971368
41	H	H1	-0.9232680	-4.7682398	-2.5826828
42	H	H2	0.7028725	-3.8605067	4.0747654
43	H	H3	-2.1176891	3.0261883	-1.3943407
44	H	H4	-2.0049155	2.2595296	0.1895730
45	H	H5	1.1968535	3.6333076	1.2214578
46	H	H6	-0.6693803	-1.3638995	-4.9550074
47	H	H7	-2.5100954	1.4026468	-4.9359315
48	H	H8	-1.9539135	3.1231705	-5.0724267
49	H	H9	-0.8049885	1.7591952	-5.4100136
50	H	H10	-1.8320482	-6.2575755	0.6857658
51	H	H11	-2.4364338	-5.7171179	-0.9374123
52	H	H12	-0.7336689	-6.3217135	-0.7496516
53	H	H13	2.1730872	-5.5597065	0.7723353
54	H	H14	2.3329767	-3.8150404	0.3231501
55	H	H15	2.7628597	-4.3672962	1.9951982
56	H	H16	0.1917101	0.1017793	5.5142449
57	H	H17	0.4800114	4.6974246	4.2011065
58	H	H18	-0.8360020	4.0795104	3.1127151
59	H	H19	-0.5009445	3.2659360	4.7051956
60	H	H20	1.9345997	0.6048311	-1.2589105
61	H	H21	3.6006658	1.1391860	-1.0078332
62	H	H22	2.6433325	0.5539576	0.3645008
63	H	H23	-0.5373262	5.2189200	0.7014831
64	H	H24	0.3858920	3.6910723	-2.3810697
65	H	H25	-0.5690101	5.0490607	-1.8026472
66	H	H26	1.1221227	4.8682395	-1.2910865

(**TS2**)

	Atom		X	Y	Z
1	C	C0	-0.6498263	-1.2473713	-2.5387629
2	C	C1	-0.4458702	-1.0811477	-1.1284659
3	C	C2	-0.4180635	0.2219266	-0.5817050
4	C	C3	-0.9442281	1.3080249	-1.3771510
5	C	C4	-1.0961777	1.1577535	-2.7431839
6	C	C5	-0.8777864	-0.1432360	-3.3542117
7	C	C6	-0.6104133	-2.5917432	-3.1448654
8	C	C7	-0.6467788	-3.7221160	-2.2421723
9	C	C8	-0.5634376	-3.5361677	-0.8990560
10	C	C9	-0.2401771	-2.2252374	-0.2790518
11	C	C10	0.1737240	-2.0425012	1.0302823
12	C	C11	0.2154801	-0.7116447	1.5910087
13	C	C12	0.0631434	0.4204769	0.7535487
14	C	C13	0.6405687	-3.1200247	1.9360429
15	C	C14	0.6840998	-2.9910392	3.2867775
16	C	C15	0.4526155	-1.7006188	3.9043405
17	C	C16	0.3729724	-0.5407186	3.0022816
18	C	C17	0.4397541	0.7442437	3.5440378
19	C	C18	0.5842801	1.8705112	2.6428733
20	C	C19	0.4504699	1.7188799	1.2777649
21	C	C20	-1.4503883	2.5375924	-0.6972744
22	C	C21	0.8465060	2.8932570	0.4230749
23	C	C22	-0.3480269	3.5432086	-0.3390670
24	O	O23	-0.6252660	-2.7108885	-4.3793476
25	O	O24	-0.9576035	-0.2254207	-4.6872246
26	O	O25	-1.4755448	2.2959391	-3.4108922
27	C	C26	-1.9234716	2.1689709	-4.7873582
28	O	O27	-0.8566268	-4.4750238	0.0383837
29	C	C28	-1.2753634	-5.7936257	-0.4152721
30	O	O29	0.4009564	-1.5305527	5.1306229
31	O	O30	1.1046611	-4.1923310	1.2370615
32	C	C31	1.6786751	-5.3015846	1.9844030
33	O	O32	0.4239243	0.9980364	4.8500288
34	O	O33	1.0250188	3.0791929	3.1599005
35	C	C34	-0.0281169	3.8307632	3.8322939
36	C	C35	2.0909401	2.6583339	-0.4425242
37	C	C36	2.6201677	1.2755409	-0.6436374
38	O	O37	2.6646166	3.6183742	-0.9121465
39	O	O38	-1.0153565	4.3870480	0.6360024
40	C	C39	0.0653356	4.3911723	-1.5323779
41	H	H1	-0.7925289	-4.6937091	-2.7081567
42	H	H2	0.9357221	-3.8020003	3.9649886
43	H	H3	-2.2108292	3.0481423	-1.3352591
44	H	H4	-2.0053287	2.2655197	0.2316264
45	H	H5	1.1907425	3.7106252	1.1489029
46	H	H6	-0.8073451	-1.2428374	-5.0270774
47	H	H7	-2.7484674	1.4552661	-4.8607983
48	H	H8	-2.2500402	3.1929671	-5.0041944
49	H	H9	-1.0830227	1.8680851	-5.4243767
50	H	H10	-1.4974361	-6.2908109	0.5377894
51	H	H11	-2.1717986	-5.7118646	-1.0384599
52	H	H12	-0.4527938	-6.2850504	-0.9424961
53	H	H13	2.0049594	-5.9658965	1.1739049
54	H	H14	2.5293801	-4.9547643	2.5804701
55	H	H15	0.9101827	-5.7720183	2.6043831
56	H	H16	0.3796083	0.0857965	5.4241181
57	H	H17	0.5133728	4.7189002	4.1749026
58	H	H18	-0.8124653	4.0832908	3.1091583
59	H	H19	-0.4140269	3.2520615	4.6780974
60	H	H20	1.9689294	0.6803035	-1.3008122
61	H	H21	3.6124265	1.3039761	-1.1225104
62	H	H22	2.7287042	0.7198920	0.2978280
63	H	H23	-0.5881485	5.2615742	0.7038174
64	H	H24	0.2511441	3.7707266	-2.4235021
65	H	H25	-0.7268570	5.0975415	-1.8098724
66	H	H26	0.9865311	4.9680306	-1.3574539

References:

(1) Al Subeh, Z. Y.; Raja, H. A.; Monro, S.; Flores-Bocanegra, L.; El-Elimat, T.; Pearce, C. J.; McFarland, S. A.; Oberlies, N. H. Enhanced production and anticancer properties of photoactivated perylenequinones. *J. Nat. Prod.* **2020**, *83*, 2490-2500.

(2) Al Subeh, Z. Y.; Waldbusser, A. L.; Raja, H. A.; Pearce, C. J.; Ho, K. L.; Hall, M. J.; Probert, M. R.; Oberlies, N. H.; Hematian, S. Structural diversity of perylenequinones is driven by their redox behavior. *J. Org. Chem.* **2022**, *87*, 2697-2710.

(3) Pocsi, I.; Prade, R. A.; Penninckx, M. J. Glutathione, altruistic metabolite in fungi. *Adv. Microb. Physiol.* **2004**, *49*, 1-76.

(4) Tirado-Rives, J.; Jorgensen, W. L. Performance of B3LYP density functional methods for a large set of organic molecules. *J. Chem. Theory. Comput.* **2008**, *4*, 297-306.
